# Phospho-JNK agonists show promising effects for the treatment of hepatocellular carcinoma

**DOI:** 10.1016/j.isci.2026.116005

**Published:** 2026-05-20

**Authors:** Woonghee Kim, Han Jin, Peipei Miao, Mehmet Ozcan, Xinmeng Liao, Mengzhen Li, Shazia Iqbal, Jihad Sebhaoui, Sajda Ashraf, Burcu Belmen, Hasan Turkez, Jan Boren, Mathias Uhlen, Xiaojing Shi, Cheng Zhang, Adil Mardinoglu

**Affiliations:** 1 Science for Life Laboratory, KTH – Royal Institute of Technology, 17165 Stockholm, Sweden; 2 Central Laboratory, Tianjin Medical University General Hospital, Tianjin 300052, P.R. China; 3 Tianjian Laboratory of Advanced Biomedical Sciences, Academy of Medical Sciences, Laboratory Animal Center, State Key Laboratory of Esophageal Cancer Prevention & Treatment, Zhengzhou University, Zhengzhou 450052, P.R. China; 4 Faculty of Medicine, Zonguldak Bulent Ecevit University, Zonguldak 67600, Turkiye; 5 Trustlife Labs Drug Research & Development Center, Istanbul 34774, Turkiye; 6 Life and Health Sciences Laboratory, FMP, Abdelmalek Essaadi University, Tetouan 93000, Morocco; 7 Department of Medical Biology, Faculty of Medicine, Atatürk University, Erzurum 25030, Turkey; 8 Department of Molecular and Clinical Medicine, University of Gothenburg, Sahlgrenska University Hospital, 41390 Gothenburg, Sweden; 9 Institute of Liver Studies, Faculty of Life Science & Medicine, King’s College London, London SE5 9RS, UK; 10 Centre for Host-Microbiome Interactions, Faculty of Dentistry, Oral & Craniofacial Sciences, King’s College London, London SE1 9RT, UK

**Keywords:** Biological sciences

## Abstract

Hepatocellular carcinoma (HCC) remains difficult to treat due to its limited targets. Hence, we introduced phosphorylated c-Jun *N*-terminal kinase (p-JNK) as an anti-HCC target protein and investigated JNK-IN-5A and six derivatives (SET135, SET156, SET158, SET159, SET171, and SET172) which stabilize p-JNK. *In vitro*, these compounds outperformed sorafenib and regorafenib, inducing stronger p53-mediated cell-cycle arrest, autophagy, apoptosis, and reduced invasiveness via JNK/c-Jun pathways. RNA-seq profiling revealed distinct mechanisms: SET135 triggered autophagic necrosis via p62/SQSTM1, while SET171 induced reactive oxygen species (ROS)-driven necrosis. Systems biology analysis confirmed their enhanced efficacy. A 7-day GLP-like rat toxicity study showed SET135 and SET171 were well-tolerated. *In vivo* study performed with 21-day treatment of SET135 or SET171 showed superior anti-tumor effects compared to sorafenib via apoptotic mechanisms in HCC-transplanted mice. These findings highlight JNK-IN-5A derivatives as promising HCC therapeutic candidates capable of inducing both apoptotic and necrotic cell death.

## Highlights

• Novel JNK-IN-5A derivatives showed anti-HCC effect via p-JNK agonism/targeting
• SET135 and SET171 induce p53-mediated apoptosis and autophagic necrosis in HCC cells
• SET135 and SET171 drive necrotic tumor cell death via p62/SQSTM1 and lipid peroxidation
• SET135 and SET171 outperform sorafenib in suppressing tumor growth *in vivo*

## Graphical abstract

**Figure:**
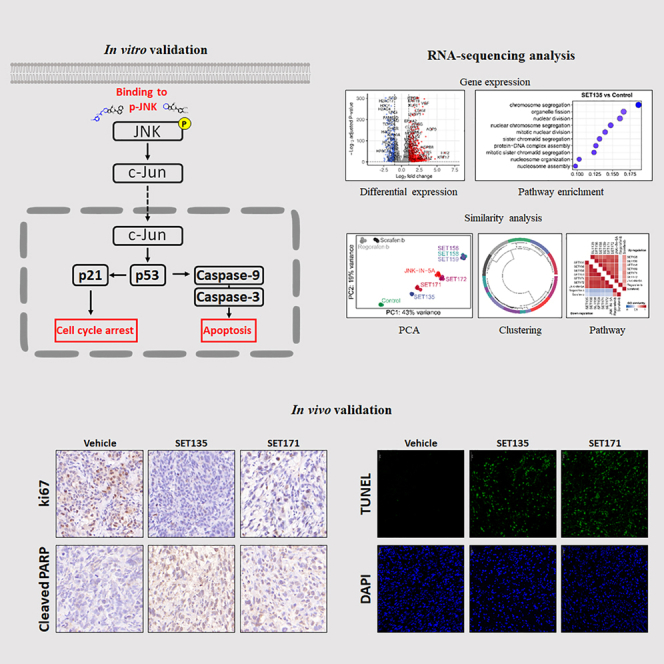

## Introduction

Primary liver cancer ranks as the sixth most prevalent cancer globally and holds the third position in terms of mortality. Hepatocellular carcinoma (HCC) represents the predominant form of primary liver cancer.^1^ The significant risk factors for developing HCC include chronic infection with hepatitis B virus (HBV) or hepatitis C virus (HCV), heavy alcohol consumption, metabolic dysfunction-associated steatohepatitis (MASH), and exposure to aflatoxins or pyrrolizidine alkaloids. These risk factors underscore the complex etiology of HCC, highlighting the importance of understanding and mitigating these risks in its prevention and management.^2^^,^^3^^,^^4^

Patients diagnosed with early-stage HCC are typically advised to undergo surgical liver resection or liver transplantation. Those in the intermediate stage or unable to undergo surgery may be considered for chemotherapy.^5^ However, the application of chemotherapy in treating liver cancer faces significant challenges, including a high rate of chemoresistance.^6^ This resistance is partly due to the liver’s fundamental role in filtering foreign substances from the bloodstream, which can limit the effectiveness of systemic chemotherapy. To enhance the efficacy of chemotherapy, medical professionals often employ regional chemotherapy techniques such as hepatic artery infusion (HAI) or transarterial chemoembolization (TACE).^7^ These methods deliver chemotherapy directly to the liver, aiming to increase drug concentration at the tumor site while minimizing systemic exposure and side effects. Multi-target kinase inhibitors, like sorafenib and regorafenib, have become widely used in the treatment of liver cancer.^8^^,^^9^ These drugs reduce angiogenesis, the process of new blood vessel formation, by inhibiting key receptors such as the vascular endothelial growth factor receptor-2 (VEGFR-2) and the platelet-derived growth factor receptor (PDGFR), which are found in endothelial cells. Additionally, they target cancer cell growth by inhibiting specific kinases, such as Raf-1 and c-Kit.^10^^,^^11^ This multifaceted approach inhibits tumor growth and metastasis by simultaneously disrupting angiogenesis and cellular proliferation pathways.

Despite the integration of multi-target kinase inhibitors with chemoembolization techniques in the treatment of HCC, the primary objectives of chemotherapy remain tumor size reduction and anti-angiogenesis.^12^ To develop more effective anti-HCC agents, researchers are focusing on designing new drug candidates that target specific proteins.^13^ In our previous research, we employed systems biology methodologies, along with co-expression network analysis, to identify pyruvate kinase L/R (PKLR) as a potential therapeutic target for HCC patients.^13^^,^^14^^,^^15^ Following this discovery, we conducted a computational drug repositioning study to identify small molecules that could inhibit or modulate PKLR gene expression. We identified JNK-IN-5A as a potent inhibitor of PKLR expression, exhibiting cytotoxic effects on the HepG2 human liver cancer cell line using *in vitro* assays.^16^ JNK-IN-5A is a small-molecule inhibitor originally developed to target c-Jun *N*-terminal kinases (JNKs). Targeting JNKs may present a promising strategy for HCC patients with sorafenib resistance. The lack of response to sorafenib in HCC has been associated with the activation of JNKs, which is linked to the increased expression of the cancer stem cell marker CD133.^10^ This finding suggests that inhibitors like JNK-IN-5A could offer a new therapeutic target for HCC patients, particularly those who exhibit resistance to current frontline treatments such as sorafenib.

In this study, we selected six JNK-IN-5A derivatives that showed the highest toxicity in our previous analysis.^17^ We first performed *in vitro* experiments to test the anti-HCC efficacy of selected derivatives, including SET135, SET156, SET158, SET159, SET171, and SET172. We observed that these compounds demonstrated significant activation of the p53 tumor suppressor pathway, leading to cell-cycle arrest, enhanced autophagy, and mitochondrial apoptosis. Notably, the most potent compounds, SET135 and SET171, induced necrotic cell death alongside apoptosis. SET135 was found to trigger p62-involved autophagic necrotic cell death, whereas SET171 induced extreme levels of reactive oxygen species (ROS), leading to lipid peroxidation and subsequent necrotic cell death. Second, we revealed the MoA of JNK-IN-5A derivatives by conducting global transcriptomics analysis and employing a systems biology-based approach. We performed bioinformatics analysis to elucidate the distinct pathways that are affected by these compounds. Furthermore, we conducted a 7-day GLP-like toxicity study in rats to assess the safety of these compounds. Finally, a 21-day mouse study suggested that SET135 and SET171 potently induce apoptotic tumor death in the transplanted Huh7 cells, showing a superior therapeutic effect and stability compared to sorafenib. This comprehensive approach underscores the unique MoA of each derivative, highlighting their potential as targeted therapies for HCC. Our findings highlight the therapeutic potential of these compounds in treating HCC, particularly by targeting specific cellular pathways to induce cancer cell death.

## Results

### JNK-IN-5A derivatives induce apoptotic cell death, cell-cycle arrest, and autophagy via activation of phospho-JNKs pathway

**Figure 1:**
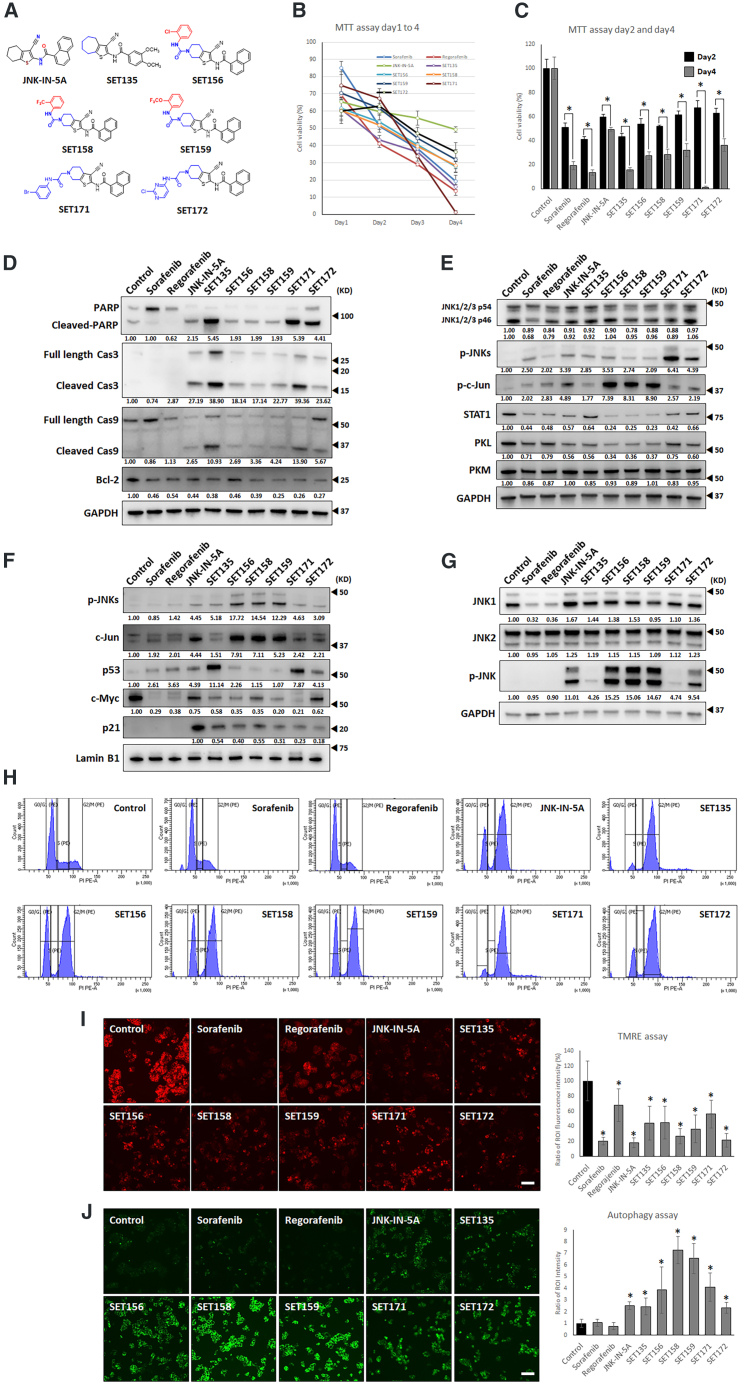
Six JNK-IN-5A derivatives induce apoptosis, cell-cycle arrest, and autophagy via p53 pathway (A) Structure of the JNK-IN-5A and six derivatives. (B and C) Time-dependent cell viability assay (MTT) for day 1 to day 4 at 10 μM drug concentration. (D) Apoptosis pathway proteins western blot analysis of whole lysate. (E) p-JNK pathway proteins western blot analysis of whole lysate. (F) Nuclear protein western blot analysis. (G) Cellular thermal shift assay (CETSA). Cells were treated at 20 μM for 2 h and had heat shock at 55°C for 3 min. (H) FACS analysis with PI staining. (I) Mitochondrial membrane potential assay. 2 μM TMRE staining on 10 μM drug-treated HepG2 cells for 1 day. Scale bars, 100 μm. (J) Autophagy assay. Autophagic vacuoles were stained with GFP fluorescence. GFP fluorescence signals were measured by region of interest (ROI) using ImageJ program. Scale bars, 100 μm. Data are represented as mean ± SD. Significance was tested using *t* test (∗*p* < 0.05).

A structure-guided design strategy was employed to develop next-generation JNK-IN-5A derivatives. The chemical reactivity and modifiable regions of the parent scaffold were analyzed, identifying three primary sites for optimization: the naphthalene moiety, cyclohexane ring, and amide linker. Each site was systematically substituted with alternative functional groups to explore structure-activity relationships (SAR) and improve target engagement with PKLR. Molecular docking studies were performed using the crystal structure of JNK in complex with compound JNK-5e to guide structural refinement.^18^ Designed molecules were ranked according to docking S-scores, root-mean-square deviation (RMSD) values, and the retention of key hydrogen-bonding and hydrophobic interactions observed in the JNK-5e complex.^17^ Based on computational prioritization, 37 derivatives were synthesized via systematic chemical derivatization of JNK-IN-5A, utilizing amide coupling, heteroaryl substitution, and selective alkylation reactions under optimized conditions. Thirty-seven JNK-IN-5A derivatives were screened for their anti-HCC effect on HepG2 cells via the cell viability assay^17^^,^^19^ (Figure S1A). We selected six derivatives based on their potent toxicity against HepG2 human liver cancer cell lines, including the top four most toxic compounds, SET159, SET158, SET171, and SET156, along with two additional drugs, SET135 and SET172, which demonstrated high toxicity after 2 and 4 days of treatment. The structures of these six compounds are presented in Figure 1A.

We reassessed the anti-HCC effects of JNK-IN-5A and the six derivatives alongside the FDA-approved liver cancer drugs, including sorafenib and regorafenib, using the HepG2 cells (Figure 1). Initially, we tested the cell viability using the MTT assay at a concentration of 10 μM for up to 4 days (Figure 1B). The compounds significantly reduced cellular viability: sorafenib (51% ± 3.6), regorafenib (41.1% ± 2.4), JNK-IN-5A (59.7% ± 2.3), SET135 (43.1% ± 2.7), SET156 (53.7% ± 4.3), SET158 (51.9% ± 0.8), SET159 (61.6% ± 2.9), SET171 (67.4% ± 5.7), and SET172 (62.7% ± 4.0) at day 2 (Figure 1C). Sorafenib (19.2% ± 3.4) and regorafenib (13.4% ± 2.3) demonstrated a time-dependent decrease in cell viability at day 4. Notably, SET171 showed the lowest cell viability at 1.1% ± 1.1 on day 4, while SET135 also exhibited a strong cell viability inhibition effect at 15.9% ± 1.4 (Figures 1B and 1C). Both SET135 and SET171 significantly induced necrotic cell death, as evidenced by propidium iodide (PI) staining (Figure S1D). The IC_50_ values of the compounds were observed (Figure S1E). Based on IC50 values, all compounds were treated at 10 μM to observe fully affected HepG2 cells by compounds. These data suggest that JNK-IN-5A and six derivatives may operate through a different MoA compared to sorafenib and regorafenib, providing a promising avenue for future drug development for the treatment of HCC. We examined the expression of proteins associated with the apoptosis pathway using western blot analysis. This was conducted on HepG2 cells treated for 2 days with 10 μM compounds (Figure 1D). JNK-IN-5A and six derivatives showed strong apoptosis protein expression, cleaved PARP, cleaved caspase3, and cleaved caspase9 than sorafenib and regorafenib. HepG2 cells treated with SET135 and SET171 exhibited a markedly higher increase in cleaved PARP (5.45- and 5.39-fold, respectively). Additionally, full length and active caspase 3 levels were significantly elevated in cells treated with SET135 (38.90-fold) and SET171 (39.36-fold). Active caspase3 enzymatic activity assay confirmed again SET135 and SET171 (Figure S1B) significantly increased caspase3 activation. SET135 showed 0.71-fold caspase3 activity to docetaxel and SET171 shoed same level as caspase3 activity than docetaxel. The anti-apoptotic protein Bcl-2, which inhibits the release of mitochondrial cytochrome,^20^ was reduced following treatment with all compounds.

We also evaluated these suspected target proteins expression of JNK-IN-5A and six derivatives (Figure 1E). All compounds resulted in a relatively similar decrease in JNK1/2/3 p54 without significance. Conversely, the expression levels of JNK1/2/3 p46 were reduced by sorafenib (0.68-fold), regorafenib (0.79-fold), and SET171(0.89-fold) significantly. However, JNK-IN-5A, SET135, SET156, SET158, SET159, and SET172 were not significantly altered. STAT1, which has been reported as the regulator of PKL expression in liver cells through the JNKs pathway,^21^ was significantly reduced in all drug-treated groups. PKL expression notably decreased in cells treated with JNK-IN-5A and six derivatives. Interestingly, the protein expression level of another pyruvate kinase muscle (PKM) isoform has not been affected compared to that of PKL. These findings suggest a specific inhibitory effect of the compounds on PKL compared to PKM, potentially decreasing the side effects led by the PKM inhibition on other healthy tissues and contributing to their MoA against HCC. We further measured the protein expression levels of the activated form of JNKs, phosphorylated JNKs (p-JNKs), and their downstream transcription factor phosphorylated c-Jun (p-c-Jun). Interestingly, we observed that JNK-IN-5A and its derivatives showed significantly increased p-JNKs and p-c-Jun. Compounds that activate p-JNKs and p-c-Jun have been shown to have an anti-cancer effect, which facilitates the TNF-α pathway.^22^^,^^23^ JNK-IN-5A has been previously documented to induce cell-cycle arrest and apoptosis through the p53 and p21 pathways in pancreatic cancer and T cell leukemia.^24^^,^^25^ In addition, its downstream protein, c-Jun, has been identified as an antagonist of p53 in liver cancer.^26^ With this in mind, we performed cellular thermal shift assay (CETSA) to find out binding affinity to JNKs and observed nuclear translocated p-JNKs, c-Jun, and anti-cancer transcription factors, p53, c-Myc, and p21 (Figures 1F and 1G).

HepG2 cells treated with 10 μM of JNK-IN-5A and derivatives for 2 days showed a significant protein expression increase in nuclear-localized p-JNKs and c-Jun (Figure 1F). Nuclear p53, which also showed as TNF-α, induced an increase in apoptotic transcription factor^23^ with treatment of JNK-IN-5a and six derivatives. SET135 (11.14-fold) and SET171 (7.87-fold) induced distinctive p53 nuclear localization. In addition, the expression of the proto-oncogene c-Myc,^27^ which is transcriptionally repressed by p53, was reduced following the treatment with these six derivatives. Sorafenib and regorafenib also decreased nuclear c-Myc expression, consistent with the previous studies.^28^^,^^29^ The six derivatives and JNK-IN-5A also significantly reduced c-Myc levels, with SET171 demonstrating the most substantial decrease compared to sorafenib and regorafenib. Interestingly, the p53-dependent tumor suppressor protein p21 was found to be specifically localized in the nucleus in the groups treated with the six derivatives and JNK-IN-5A, but not in the groups treated with sorafenib or regorafenib. This indicates that these six derivatives and JNK-IN-5A may uniquely influence the nuclear translocation of p21,^30^ suggesting a binding to p-JNKs and nuclear translocation, which leads TNF-α pathway from the other therapies with sorafenib and regorafenib.

JNK-IN-5A and six derivatives showed strong binding to p-JNK rather than JNK1 and JNK2 in HepG2 cells (Figure 1G). Sorafenib has been reported to regulate p-JNK via interaction with JNKs.^31^ We found that sorafenib and regorafenib binds to JNK1 but not JNK2 and p-JNK. However, CETSA and nuclear translocation analyses indicate that JNK-IN-5A and its derivatives preferentially stabilize and engage p-JNK in HepG2 cells, suggesting enhanced interaction with the activated JNK pool compared to sorafenib and regorafenib. Interaction between JNK-IN-5A derivatives and p-JNK provide additional insight into their potential for HCC treatment.

The p53 tumor suppressor protein is widely recognized for its roles in inducing apoptosis, cell-cycle arrest, and autophagy.^32^^,^^33^^,^^34^^,^^35^ Studies suggest that both p53 and p21 are crucial for maintaining cell viability, with IC_50_ assays indicating that JNK-IN-5A and six derivatives exert a robust cell-cycle arrest effect. To verify this, we conducted PI fluorescence-activated cell sorting (FACS) analysis. PI staining and FACS analysis confirmed significant G2/M cell-cycle arrest in cells treated with the six compounds and JNK-IN-5A (Figure 1H; Table 1). In contrast, sorafenib and regorafenib did not alter the cell-cycle distribution significantly compared to the control group. Histogram plots revealed that JNK-IN-5A, SET156, SET158, SET159, and SET172 maintained 20%–30% of cells in the G0/G1 phase and 60%–70% in the G2/M phase. SET135 and SET171 induced a more pronounced cell-cycle arrest, with approximately 5%–6% of cells in G0/G1 and around 89% in the G2/M phase.

**Table 1:** Cell population on PI FACs analysis

	Control	Sorafenib	Regorafe-nib	JNK-IN-5A	SET135	SET156	SET158	SET159	SET171	SET172
**G0/G1**	62.4	62.2	70.5	22.7	5.5	30.1	31.6	30.5	6.6	16.7
**S**	15.4	10.1	9.7	4.4	2.0	2.3	3.5	3.3	3.6	2.7
**G2/M**	16.2	19.5	14.9	67.3	**79.9**	60.8	60.3	62.4	**79.1**	73.2

JNK-IN-5A and six derivatives induce G2/M cell-cycle arrest. SET135 and SET171 demonstrated the most significant cell cycle arrest potency, nearly 80% G2/M phase accumulation, which represents stronger effect than other compounds and FDA-approved anti-HCC drugs.

Bcl-2, a protein downstream of JNKs involved in the apoptotic pathway,^36^ has been reported to facilitate mitochondrial apoptosis when phosphorylated.^24^ To assess mitochondrial function during apoptosis, we examined mitochondrial membrane potential using TMRE staining (Figure 1I). HepG2 cells treated with 10 μM of sorafenib and regorafenib for 1 day showed a decrease in mitochondrial membrane potential, as previously reported.^37^ Similarly, JNK-IN-5A, SET135, SET156, SET158, SET159, SET171, and SET172 also significantly decreased mitochondrial membrane potential. Furthermore, JNK and Bcl-2 are known to participate in autophagic cell death.^38^^,^^39^ We performed autophagy assays on HepG2 cells treated with 10 μM of the compounds for 1 day (Figure 1J). Notably, while sorafenib and regorafenib did not induce autophagy, JNK-IN-5A and six derivatives markedly increased autophagy GFP signals. These findings collectively suggest that these compounds induce apoptotic cell death through a multifaceted mechanism involving strong cell-cycle arrest, diminished mitochondrial membrane potential, and pronounced autophagy, all of which are mediated via the p53 pathway. The implications of these effects highlight the potential of these compounds as multifunctional agents in HCC therapy.

We have shown that JNK-IN-5A and six derivatives induce apoptotic cell death, cell-cycle arrest, and autophagy through the activation of the p53 tumor suppressor pathway via p-JNKs activation. This mechanism highlights the compounds that engage a critical regulatory pathway in cell biology, exploiting the role of the p53 protein in maintaining cellular integrity by triggering responses to DNA damage, stress signals, and aberrant growth signals. By activating this pathway, the JNK-IN-5A and six derivatives effectively promote the removal of damaged or cancerous cells, providing a targeted approach to HCC therapy that harnesses the body’s natural defences against tumor progression.

### SET135 and SET171 reprogram the necrotic cell death

**Figure 2:**
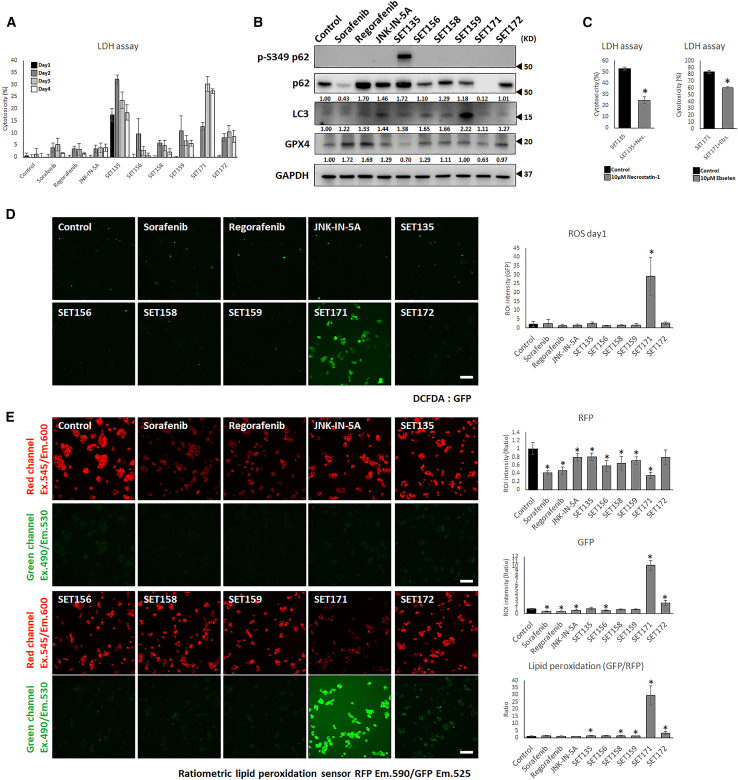
SET135 and SET171 induce necrotic cell death by necroptosis and ferroptosis (A) Time-dependent LDH assay. Day 1 to day 4. (B) Western blot analysis for cellular expression protein levels of autophage and peroxidation. (C) LDH assay. 10 μM SET135 with 10 μM Necrostatin-1 and 10 μM SET171 with 10 μM Ebselen for 2 days treatment. (D) Cellular ROS assay. 20 μM DCFDA staining on 10 μM drug-treated HepG2 cells for 1 day. Scale bars, 100 μm. (E) Lipid peroxidation assay. Ratiometric lipid peroxidation sensor-stained peroxidised lipid. Fluorescence intensity was measured on region of interest (ROI) using ImageJ. Scale bars, 100 μm. Data are represented as mean ± SD. Significance was tested using *t* test (∗*p* < 0.05).

In our detailed investigation of the effects of the six derivatives on cell death mechanisms, we monitored the progression of necrotic cell death for up to 4 days. Notably, treatment with 10 μM SET135 and SET171 induced pronounced cytotoxicity from day 1 to day 4 (Figure 2A). As previously mentioned, SET135 also triggers apoptosis (Figure 1D). The concurrent induction of apoptosis and necrosis observed in this study is reminiscent of the effects reported for Tanshinone IIA treatment on HepG2 cells, a process termed necroptosis.^40^ Necroptosis is characterized as programmed cell necrosis, distinguishing it from apoptosis in several key ways.^41^^,^^42^ Unlike apoptosis, during necroptosis, the protein p62/SQSTM1 recruits autophagic proteins to form a necrosome, leading to necrotic cell death.^43^ To further understand this process in the context of SET135 treatment, we analyzed the expression of p62 (SQSTM1) and its phosphorylated form, phospho-p62 (S349)—an activated state of p62—using western blot analysis (Figure 2B). Treatment of HepG2 cells with 10 μM SET135 for 2 days resulted in significantly elevated levels of both p62 and p-S349 p62. We found LC3, the central protein in autophagy pathway, relatively increased by SET135 but not significant. However, SET135 only increased p62, p-S349 p62, and LC3 at the same time. This observation suggests that SET135 may activate necroptosis in HepG2 cells through mechanisms involving both the upregulation of p62, p-S349 p62, and LC3 contributing to the formation of the necrosome complex and showing necrotic cell death. For further confirmation of necroptosis induced necrosis, we co-treated Necrostatin-1^44^ with SET135 (Figure 2C). 10 μM Necrostatin-1 co-treated with SET135 showed significant reduced necrotic cell death (52.9%–24.6%).

Oxidative stress can induce cell death through multiple pathways, including mitochondrial damage via caspase-3 activation, DNA damage through cleaved PARP, and the initiation of autophagy.^45^^,^^46^^,^^47^ To understand the mechanism behind cell death induced by JNK-IN-5A and six derivatives, we assessed cellular ROS levels in HepG2 cells treated with 10 μM of the compounds for 1 day. Cells were stained with the ROS detector 2′,7′-dichlorodihydrofluorescein diacetate (DCFDA). The fluorescence image analysis showed that SET171 significantly increased cellular ROS (Figure 2D). Notably, SET171 was associated with pronounced late necrotic cell death from days 3 and 4 (Figure 2A).

Previous studies have demonstrated that elevated cellular ROS can induce lipid peroxidation, leading to membrane rupture and necrotic cell death, a process referred to as ferroptosis.^48^^,^^49^ To confirm lipid peroxidation, we employed a ratiometric lipid peroxidation sensor staining (Figure 2E). This sensor stains cellular lipids with RFP fluorescence, which is replaced by GFP fluorescence upon lipid peroxidation. SET171 exhibited a statistically significant increase in the GFP/RFP ratio, indicating lipid peroxidation, unlike other compounds tested. We also measured the expression level of glutathione peroxidase 4 (GPX4), a crucial enzyme that mitigates lipid peroxidation and plays a key role in preventing ferroptosis.^50^ HepG2 cells treated with sorafenib and regorafenib showed significantly higher GPX4 expression compared to the control (Figure 2B), while treatments with JNK-IN-5A, SET156, SET158, SET159, and SET172 resulted in similar or slightly increased GPX4 levels (1.00–1.29-fold). In contrast, SET135 and SET171 showed a significantly decrease in GPX4 expression levels (0.70-fold and 0.63-fold, respectively).

We also tested ferroptosis inhibitor Ebselen^51^ with SET171. Not as much as necrosis inhibition effect of Necrostatin-1 with SET135, 10 μM ebselen significantly decreased necrotic cell death, 83.7%–60.5%, by SET171 (Figure 2C).

These results suggest that SET171 induces ferroptosis through mechanisms involving increased cellular ROS, lipid peroxidation, and reduced GPX4 expression.

The ability of SET135 and SET171 to simultaneously induce apoptosis and necrosis. SET135 and SET171 showed necroptosis and ferroptosis features. However, it is not clear that how much necrotic cell death affect to anti-HCC effect and how related to apoptotic cell death. Despite unclear specific pathway between apoptosis and necrosis, SET135 and SET171 represent a promising approach to overcome resistance to apoptosis in cancer cells, highlighting their potential as therapeutic agents in HCC treatment strategies.^52^

### JNK-IN-5A derivatives inhibit cancer invasion and mobility in HepG2 cells

**Figure 3:**
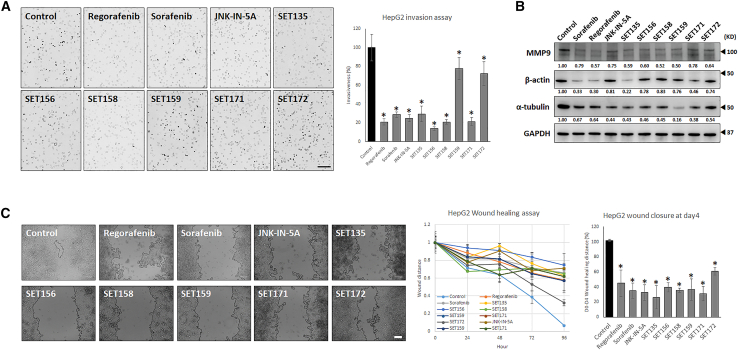
Six JNK-IN-5A derivatives inhibits cancer cell invasion and motility (A) Invasion assay. Cells were treated at 1 μM of each drug and observed after 24 h invasion assay. Scale bars, 100 μm. (B) Cell invasion and migration involved proteins western blot analysis of whole lysate. (C) Wound healing assay. Cancer cell motility was measured for 4 days with 1 μM of each drugs-treated HepG2 cells. Scale bars, 100 μm. Data are represented as mean ± SD. Significance was tested using *t* test (∗*p* < 0.05).

We assessed the impact of the compounds on the invasiveness and motility of HepG2 cells (Figure 3). Invasion assay conducted with a concentration of 1 μM revealed that regorafenib, sorafenib, JNK-IN-5A, and the six derivatives substantially reduced the invasiveness of HepG2 cells. Specifically, regorafenib reduced invasiveness by 21.0% ± 3.1, sorafenib by 28.7% ± 2.9, JNK-IN-5A by 24.9% ± 3.8, SET156 by 14.0% ± 1.9, SET158 by 20.8% ± 2.4, SET159 by 77.8% ± 11.5, SET171 by 21.4% ± 4.3, SET135 by 29.5% ± 8.3, and SET172 by 72.3% ± 12.6 (Figure 3A). Western blot analysis revealed that sorafenib, regorafenib, JNK-IN-5A, and the six derivatives led to a decrease in MMP9 levels (Figure 3B), a matrix metalloproteinase implicated in cancer invasion and metastasis. Furthermore, the expression levels of proteins involved in cancer cell migration and motility, such as α-tubulin^53^^,^^54^^,^^55^ and β-actin,^56^^,^^57^ were significantly reduced.

Additionally, a wound-healing assay was performed to evaluate the effects of the drugs on cancer cell motility. After achieving a confluent state, a wound was created in the HepG2 cell layer using a pipette tip, and the wound width was measured through image analysis until the control HepG2 cells closed the wounded area by day 4 (Figure 3C). The results showed that all tested compounds delayed wound healing compared to untreated HepG2 cells, which achieved 100% wound closure by day 4. Specifically, regorafenib delayed wound healing by 44.8% ± 17.4, sorafenib by 35.0% ± 9.9, JNK-IN-5A by 32.7% ± 10.4, SET156 by 26.2% ± 15.3, SET158 by 39.5% ± 6.1, SET159 by 35.5% ± 2.6, SET171 by 36.4% ± 14.0, SET135 by 31.2% ± 9.4, and SET172 by 60.7% ± 5.0. These findings suggest that the tested drugs effectively impair the invasiveness and motility of HepG2 cells, highlighting their potential as therapeutic agents in limiting HCC progression and metastasis.

### JNK-IN-5A derivatives show unique transcriptional profiles in HepG2 cells

**Figure 4:**
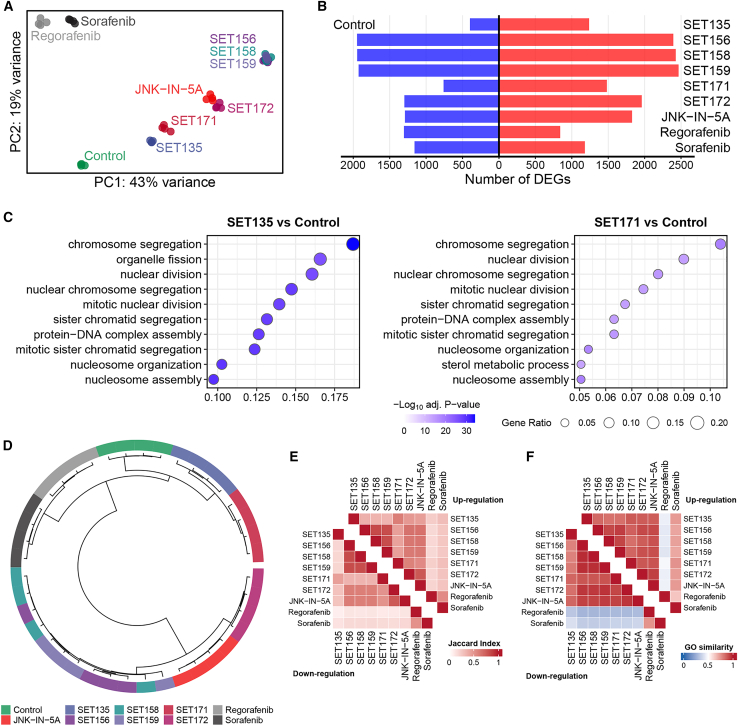
RNA-seq analyses of HepG2 cells treated by the six JNK-IN-5A derivatives and known HCC drugs (A) PCA plot shows the distribution of HepG2 cells treated by the JNK-IN-5A and six derivatives, sorafenib and regorafenib. (B) The number of differentially expressed genes in different treatment groups. (C) GSOA of downregulated genes induced by the treatment with SET135 and SET171. *p* values were adjusted by the Benjamini-Hochberg procedure. (D) Hierarchical clustering of samples based on gene expression profiles. (E) The Jaccard index between DEGs from different treatments. (F) GO semantic similarity between significant GO terms from different treatments.

To elucidate the changes in gene expression and biological pathways induced by drug treatment, we conducted RNA sequencing (RNA-seq) analysis on HepG2 cells treated with JNK-IN-5A, six derivatives, sorafenib and regorafenib. The analysis revealed distinct transcriptional profiles for each treatment group compared to the control, as shown in Figure 4A. Notably, the transcriptional responses to sorafenib and regorafenib were more similar to each other than to the responses elicited by the six derivatives and JNK-IN-5A. This suggests that JNK-IN-5A and six derivatives may activate different mechanistic pathways from those targeted by sorafenib and regorafenib. Among the JNK-IN-5A derivatives, SET172 displayed the closest transcriptional similarity to JNK-IN-5A. SET156, SET158, and SET159 clustered closely together, indicating similar transcriptional changes, while SET135 and SET171 constituted another distinct group.

In terms of differentially expressed genes (DEGs), sorafenib and regorafenib induced a smaller number of DEGs compared to the other treatments. In contrast, SET135 and SET171 exhibited a moderate number of DEGs, highlighting their potential for inducing significant transcriptional changes (Figures 4B and S2). Gene ontology (GO) analysis and gene set overrepresentation analysis (GSOA) of the DEGs revealed a significant association with cell-cycle regulation. Specifically, downregulated DEGs by SET135 and SET171 were closely linked to cell-cycle processes, with SET135 showing a more pronounced effect than SET171 and the other four derivatives (Figures 4C and S3). This RNA-seq analysis underscores the unique impacts of JNK-IN-5A and six derivatives on HepG2 cells, particularly in modulating cell-cycle-related genes, and distinguishes their effects from those of sorafenib and regorafenib, suggesting a MoA for these compounds in HCC treatment.

Hierarchical clustering analysis of gene expression profiles revealed that the compounds could be categorized into three distinct clusters, in addition to the control group. These clusters include sorafenib and regorafenib as one group, SET135 and SET171 as another, and the last cluster comprising the remaining JNK-IN-5A and four derivatives (Figure 4D). To further assess the similarity between DEGs resulting from various treatments, we employed the Jaccard index and semantic similarity analyses^58^ based on significant GO biological pathways associated with these DEGs. The outcomes of these analytical approaches were congruent, demonstrating that sorafenib and regorafenib exhibited less similarity to JNK-IN-5A and six derivatives (Figures 4E and 4F). This finding aligns with the results obtained from principal component analysis (PCA) and clustering analysis (Figures 4A–4E), reinforcing the notion that JNK-IN-5A and six derivatives potentially engage distinct biological mechanisms compared to sorafenib and regorafenib. Within the group of JNK-IN-5A and three derivatives, including SET156, SET158, and SET159, there was a close relationship, indicating a high degree of similarity among their effects. These compounds also showed a notable resemblance to JNK-IN-5A, suggesting shared pathways or targets that might underlie their therapeutic actions.

**Figure 5:**
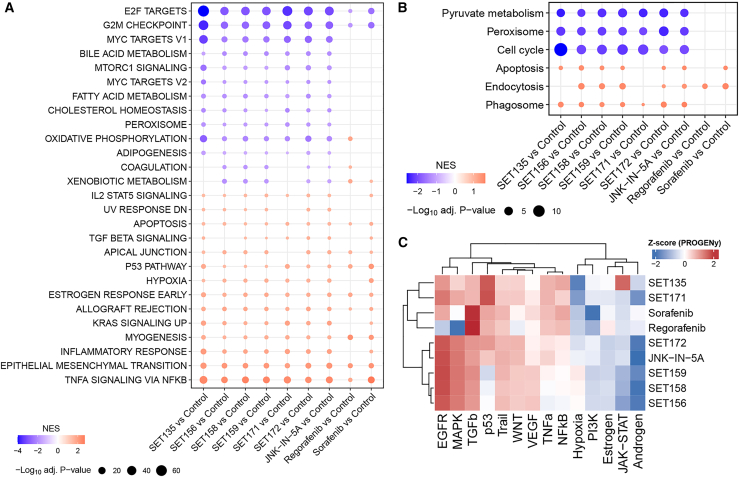
Altered pathways in HepG2 cells treated by the six JNK-IN-5A derivatives and known HCC drugs (A) GSEA of representative hallmark gene sets in different treatment groups compared to control. (B) GSEA of representative KEGG pathways in different treatment groups compared to control. For (A) and (B), NES: normalized enrichment score; *p* values were adjusted by the Benjamini-Hochberg procedure. (C) Activity scores (presented as *Z* score) of the 14 cancer-related PROGENy pathways for the nine treatment groups in this study.

To thoroughly assess the pathways disrupted by the treatments, gene set enrichment analysis (GSEA) was conducted using hallmark gene sets from the Molecular Signatures Database (MSigDB)^59^ and the Kyoto Encyclopedia of Genes and Genomes (KEGG) pathway.^60^ Figure 5A illustrates that all six derivatives more effectively inhibited cell-cycle-related pathways, such as E2F targets and the G2M checkpoint, compared to sorafenib and regorafenib. Among these, SET135 was identified as the most potent inhibitor, closely followed by SET171. Moreover, all treatments were found to upregulate apoptosis signaling. Notably, JNK-IN-5A and six derivatives exhibited liver-specific effects, specifically inhibiting bile acid metabolism and fatty acid metabolism. Additionally, oxidative phosphorylation (OXPHOS) was uniquely suppressed by treatments involving the JNK-IN-5A and six derivatives. These findings underscore that while all treatments robustly hinder the cell cycle, the JNK-IN-5A and six derivatives offer additional liver-specific actions that contribute to their efficacy in inhibiting cell growth. This suggests a broader spectrum of therapeutic effects, particularly relevant for liver-related conditions, underscoring the potential of these compounds for targeted liver cancer therapy.

The findings derived from the analysis of hallmark gene sets were further corroborated by KEGG pathway analysis. Illustrated in Figure 5B, pyruvate metabolism was significantly suppressed in all treatments involving the JNK-IN-5A and six derivatives. Moreover, a pronounced inhibition of the cell cycle was observed with these treatments, notably with SET135 displaying the most substantial effect. Additionally, there was significant suppression of the peroxisome pathway, which may be related to autophagy, in treatments with the JNK-IN-5A and six derivatives, unlike in those with sorafenib and regorafenib. When evaluating pathways associated with programmed cell death, such as apoptosis, endocytosis, and phagosome, the analysis did not reveal a clear distinction between the effects of the six derivatives, sorafenib and regorafenib. This suggests that while the JNK-IN-5A and six derivatives uniquely impact specific metabolic and cell-cycle pathways, their influence on programmed cell death pathways may share similarities with that of sorafenib and regorafenib. These insights suggest that the unique therapeutic potential of the JNK-IN-5A and six derivatives could be attributed to their specific mechanisms of action on metabolic regulation and cell-cycle control, rather than a fundamentally different approach to inducing programmed cell death.

Using PROGENy, a computational algorithm designed to infer the activity of 14 cancer-related signaling pathways from gene expression data,^61^ we observed that SET135 and SET171 significantly activated the p53 signaling pathways, confirming the findings shown in Figure 1. Furthermore, while sorafenib and regorafenib showed a high level of activation of the TGFβ pathway, such activation was not observed in treatments with the six derivatives. This suggests a different functional profile of these compounds compared to the two FDA-approved cancer drugs.

Our comprehensive analyses using HepG2 cells indicate that although all treatments are capable of inhibiting cancer cell growth, with SET135 and SET171 being the most effective of the six derivatives, JNK-IN-5A likely possesses unique and liver-specific effects that distinguish it from sorafenib and regorafenib. This distinction suggests that JNK-IN-5A and six derivatives might offer alternative therapeutic mechanisms, potentially providing new avenues for the treatment of HCC, particularly by targeting pathways such as p53, which are crucial in the regulation of cancer cell survival and death.

### Normal liver cell line and GLP-like toxicity study in rats supporting the safety of the compounds

We have checked toxicity to normal cells on the THLE-2 non-tumorigenic human liver epithelial cell line *in vitro* (Figure S1C). All compounds were treated at 5 μM concentration to 5,000 cells for 2 days. JNK-IN-5A and derivatives showed significantly reduced toxicity at THLE-2 cells as sorafenib and regorafenib.

Seven-day oral tolerability study was conducted in Wistar rats to evaluate the safety of SET135 and SET171 at three dose levels: 30 mg/kg, 100 mg/kg, and 300 mg/kg body weight. The study included a vehicle control group (four males and four females) and three dose groups for each compound, with each dose group consisting of three male and three female rats. Dosing was performed once daily in the morning for 7 consecutive days.

**Figure 6:**
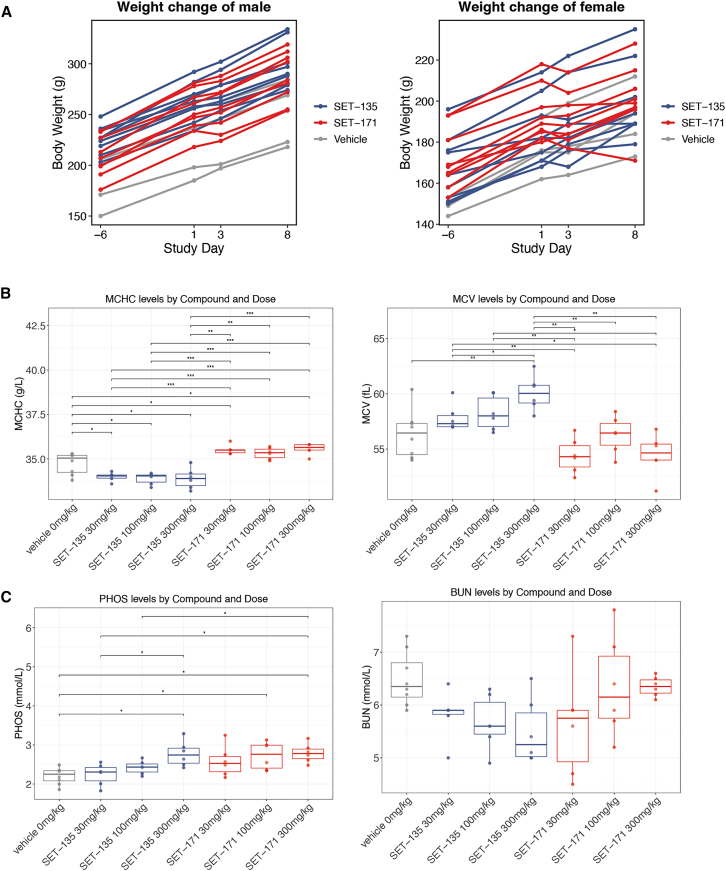
GLP-like toxicity study supporting the safety of the compounds in rats (A) Body weight changes over time across treatment groups. (B and C) Mean corpuscular hemoglobin concentration (MCHC), mean corpuscular volume (MCV), and phosphate (PHOS) levels exhibited statistically significant changes over time. Blood urea nitrogen (BUN) levels remained stable, suggesting no evidence of kidney toxicity.

No clinical adverse effects were observed in the treated animal groups. Body weight, hematology, and plasma chemistry were assessed throughout the study. We observed that the treatment with these compounds did not affect body weight (Figure 6A). While mean corpuscular hemoglobin concentration (MCHC) and mean corpuscular volume (MCV) showed statistically significant changes in some treated animals, these changes were randomly distributed across dose groups and showed no clear dose-dependent response (Figure 6B). Phosphate (PHOS) levels were slightly elevated in a dose-dependent manner in both SET135 and SET171 groups, whereas blood urea nitrogen (BUN) levels remained within the normal range, indicating no kidney toxicity (Figure 6C). These minor variations were considered incidental and unrelated to treatment. All other parameters remained within normal ranges, further supporting the safety of both compounds (Figure S6).

### JNK-IN-5A derivatives show *in vivo* anti-tumor activity

**Figure 7:**
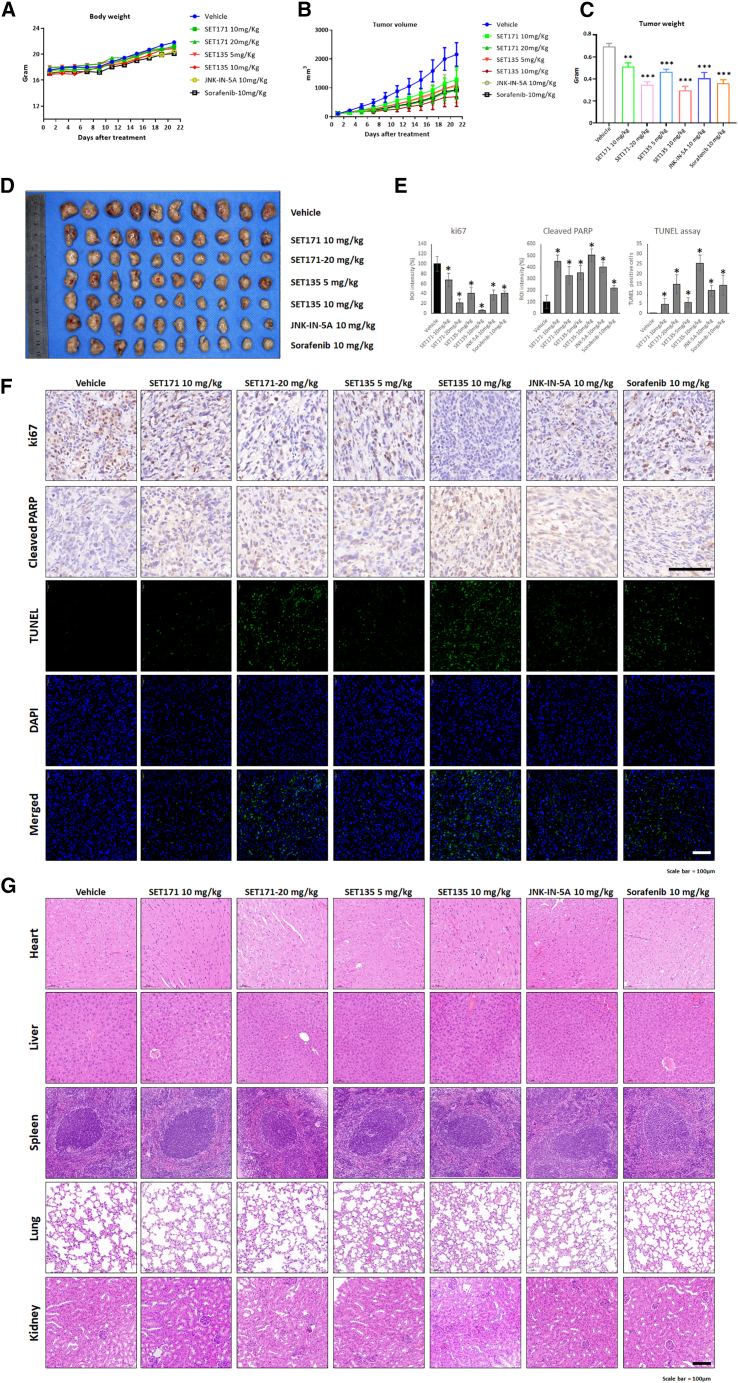
The anti-tumor efficacy of the SET135 and SET171 in Huh7 bearing animal model Huh7 cells were transplanted subcutaneously to the BALB/nude mice and subjected to SET171 (10 and 20 mg/kg), SET135 (5 and 10 mg/kg), JNK-IN-5A (10 mg/kg), sorafenib (10 mg/kg), and vehicle control. (A) Body weight changes for 21 days measured every other day. (B) Tumor volume changes for 21 days measured every other day. (C and D) Isolated tumor weights and image after 21 days treatment. (E and F) Statistic and image of IHC staining (ki67 and cleaved PARP) and fluorescence staining TUNEL positive cells. Scale bars, 100 μm. (G) Represented H&E staining images of the major organs of the mice after 21 days treatment. Scale bars, 100 μm. Data are represented as mean ± SD. Significance was tested using *t* test (∗*p* < 0.05, ∗∗*p* < 0.005, ∗∗∗*p* < 0.0005). Scale bars, 100 μm.

Our analysis indicated that both SET135 and SET171 have potent inhibitory effects on HCC cell models. Hence, we evaluated the *in vivo* anti-tumor activities of these two compounds in Huh7 HCC xenograft tumor animal models. Mice were treated with the compounds every other day for 21 days. We found that the body weights of mice in all treatment groups were not significantly different from those of the control group (Figure 7A). We observed that JNK-IN-5A, SET135, SET171, and sorafenib treatment significantly inhibited tumor growth compared to vehicle control (Figure 7B).

After 21 days of compound treatment, we performed visual inspection and weight measurement of the tumor tissues (Figures 7C and 7D). Daily administration of 10 mg/kg sorafenib decreased tumor weight to 52.27% compared to vehicle control. JNK-IN-5A at 10 mg/kg in the treated group showed a 58.6% reduction in tumor weight. SET171 at doses of 10 and 20 mg/kg treated groups markedly and dose-dependently decreased the tumor weight to 73.87% and 50.05%, respectively. On the other hand, SET135 at doses of 5 and 10 mg/kg in the treated groups showed a markedly and dose-dependently decrease in tumor weight to 67.19% and 42.65% compared to the vehicle control, respectively. We observed that 20 mg/kg SET171- and 10 mg/kg SET135-treated groups showed stronger inhibitory effects on tumor growth compared to the 10 mg/kg sorafenib-treated group.

We investigated *in situ* apoptosis using a cleaved PARP IHC, TUNEL assay and cell-cycle arrest using ki67 IHC for 21 days of compound-treated tumor tissues (Figures 7E and 7F). Consistent with overall tumor suppressive activity, we detected significantly induced cell-cycle arrest and apoptosis in all treated groups. Cell proliferation marker protein ki67 was decreased at 20 mg/kg SET171 up to 21% and 10 mg/kg SET135 up to 5.7%, respectively. SET171 and SET135 treated group also showed increased cleaved PARP IHC staining intensity to 3- to 4-fold. 10 mg/kg sorafenib- and 10 mg/kg JNK-IN-5A-treated groups induced 14.27% and 11.54% TUNEL positive cells, respectively. 10 mg/kg and 20 mg/kg SET171-treated groups showed significant and dose-dependently induced apoptosis at 4.71% and 14.84%. SET135 also showed highly increased apoptotic cells by 5.64% at 5 mg/kg dose and 25.4% at 10 mg/kg dose. Notably, the 10 mg/kg SET135-treated group showed stronger TUNEL-positive cells than the 10 mg/kg sorafenib-treated group.

Throughout the treatment period, we did not observe obvious toxicities including body weight change of mice in all treatment groups compared to the vehicle group (Figure 7A). We performed pathological and hematological examinations for six major organs, including the heart, liver, spleen, lung, and kidney tissues, based on H&E staining (Figures 7G and S7). We found that there are no significant differences in lesions or toxic-damage sites between all 21-day treated groups compared to the vehicle control. Hematological and biochemical blood tests for 21 days of compound-treated mice also showed that JNK-IN-5A, SET135, and SET171 have fewer side effects than sorafenib (Figure S7). Hematological test for WBC, RBC, HGB, MCH, MCHC, MCV, PLT, HCT ,and serum biochemical test for ALT, AST, γ-GT, TBIL, DBIL, ALP, ALB, BUN, UA, and CREA showed no significant changes in the hematological and serum biochemical parameters. These *in vivo* analyses indicated that derivatives, including SET135 and SET171, have superior or comparable anti-HCC effects and stability compared to sorafenib, a conventional HCC drug.

## Discussion

The liver, renowned for its unique roles in drug metabolism and detoxification, is a vital organ in the management of xenobiotics. HCC typically has a lower response rate to chemotherapy than many other cancers.^62^ Current clinical treatments often include multi-target kinase inhibitors such as sorafenib and regorafenib; however, the need for therapeutic options is underscored by their modest efficacy,^63^ and the development of chemoresistance in some patient^64^^,^^65^ highlights the need for therapeutic options.

In our earlier research, we identified the PKLR gene as a potential drug target for HCC treatment and proposed JNK-IN-5A as a potent inhibitor/modulator of PKLR protein expression, suggesting it as a potential therapeutic agent for HCC.^14^^,^^16^ Building on this, we have now introduced six novel drug candidates derived from JNK-IN-5A. Beyond their role in decreasing PKL expression, the JNK-IN-5A and six derivatives have demonstrated anticancer effects via p-JNKs activation and interfering TNF-α signaling pathway. JNK-IN-5A induces the expression of p53-dependent tumor suppressor proteins, decreases c-Myc levels, and leads to a significant increase in p21 expression across all treatment groups. The compounds’ ability to selectively enhance nuclear p21 expression provides strong evidence of their capacity to induce G2/M phase cell-cycle arrest. Specifically, SET135 and SET171 have shown remarkable anticancer effects, inducing both apoptotic and necrotic cell death in HepG2 cells. Moreover, these drugs significantly augment nuclear p53 expression and reduce c-Myc expression, surpassing the effects of JNK-IN-5A, sorafenib, and regorafenib. Therefore, the mechanisms by which SET135 and SET171 elicit apoptotic cell death, necroptosis, and ferroptosis merit further exploration.

SET135 has been observed to induce the overexpression of p62 and its phosphorylated form at serine 349 (p-S349 p62), which is recognized as a potent oncogenic protein.^66^ The phosphorylation of p62 at serine 349 enhances its binding affinity to the Keap1 protein, leading to the activation of the Nrf2 pathway. This pathway is well-known for its cytoprotective functions in normal cells. However, it also plays a role in tumorigenesis in pre-malignant cells, contributing to drug resistance and facilitating tumor growth. p62, which is a crucial component of protein aggregates forming Mallory-Denk bodies (MDBs) or intracellular hyaline bodies (IHBs), is considered both a prognostic and diagnostic marker for NASH and HCC.^67^^,^^68^^,^^69^ A recent study has differentiated two isoforms of p62: p62-H1, the full-length isoform, and p62-H2, which is partially devoid of the PB1 domain.^70^ These isoforms differ in their aggregation potential. Notably, p62-H2 is more predominantly expressed in human liver tissue than p62-H1. Intriguingly, in our study, the SET135 treatment group displayed a significant increase in p62-H1 expression exclusively, as shown in Figure 2B. This finding positions SET135 as a critical agent for further research into the role of p62/SQSTM1 as a target protein in disease mechanisms. The distinct expression pattern elicited by SET135 treatment provides valuable insights that could contribute to understanding the molecular underpinnings of diseases associated with p62 dysregulation.

In animal studies, SET135 and SET171 showed promising anti-HCC effects with conventional HCC drug sorafenib. For 21 days, SET135 and SET171 inhibited the transplanted Huh7 cells’ tumor growth, and the TUNEL assay revealed that they strongly induced apoptotic cell death. 10 mg/kg SET135 showed stronger tumor growth inhibition and a higher rate of apoptotic cell death. More interestingly, SET135 and SET171 did not show toxic side effects on the major five organs, heart, liver, spleen, lung, and kidney, and also hematologic and serum biochemical tests.

We repositioned the drug candidates based on their ability to modulate PKL expression levels. However, we found that siRNA-mediated inhibition of PKL did not manifest the desired anticancer effect, as shown in Figure S4, since WT HepG2 cells also express PKM2. We transfected three distinct siRNAs into HepG2 cells and, after 3 days, observed no statistically significant change in cell viability (Figure S4A). Furthermore, western blot analysis revealed that the suppression of PKL expression via siRNAs did not result in increased cleaved-PARP or decreased Bcl-2 expression levels, unlike the effects seen with JNK-IN-5A and six derivatives (Figure S4B). This suggests the presence of alternative pathways, potentially involving p53 and c-Myc, that lead to apoptosis.

Open MoA is a previously developed robust tool for elucidating the MoA of drugs by leveraging network topology and hierarchy.^21^ Utilizing drug-specific DEGs data, Open MoA constructs drug-specific subnetworks and identifies the most probable pathways from the drugs to p53 or c-Myc. All identified interactions have a high confidence score, surpassing 0.99 (Figure S5). For the JNK-IN-5A, MAPK8 or MAPK9, the known target of JNK-IN-5A, was selected as the starting point.^18^ JUN was identified as the sole intermediate target in the projected MoA, with its strong association with p53 and c-Myc supported by prior research.^26^ Regarding regorafenib, of its 18 direct downstream targets, ABL1 was pinpointed as having the highest likelihood of interacting with p53 and c-Myc. For sorafenib, among its eight downstream targets, PDGFRB and FGFR1 were deemed the most likely to influence the mechanisms leading to p53 and c-Myc, with STAT1 and SOS1 acting as intermediate proteins, respectively.

Although the predicted MoAs for sorafenib, regorafenib, and JNK-IN-5A showed significant variation, none included PKL, although all compounds inhibited PKL protein expression (Figure 1D). The intermediate proteins identified within these MoAs may serve as pivotal targets through which the drugs impact apoptosis by modulating p53 and c-Myc activation. The differing MoAs might contribute to the observed variations in cell apoptosis. To further increase our understanding and improve the efficacy of the JNK-IN-5A and six derivatives, additional *in vivo* studies are needed. These investigations should aim to fully characterize the drugs’ MoAs and their effects on cellular processes, particularly those related to p53 and c-Myc signaling pathways.

In summary, we characterized the anti-HCC effect of JNK-IN-5A and six potent derivatives, SET135, SET156, SET158, SET159, SET171, and SET172. Mechanism study revealed that six derivatives induce apoptotic cell death and cell-cycle arrest through activation of the p53 signaling pathway by activating p-JNK pathway and autophagy via mitochondrial stress by Bcl2 inhibition. SET135 and SET171 uniquely triggered necroptotic and ferroptotic pathways, indicating their multifaceted potential to overcome apoptosis resistance in HCC. Furthermore, transcriptional profiling demonstrated that these compounds modulate cell-cycle and metabolism-related pathways distinct from those targeted by sorafenib and regorafenib. SET135 and SET171 also exhibited superior *in vivo* anti-tumor efficacy compared to sorafenib, while showing safety profiles in animal model. Collectively, our findings establish SET135 and SET171 as promising multi-mechanistic therapeutic candidates for HCC treatment, providing new insights into the p-JNK pathway as a therapeutic target for HCC.

### Limitations of the study

Our study extensively showed that JNK-IN-5A and its derivatives, specifically SET135 and SET171, induces stronger apoptotic cell death compare to conventional drugs, sorafenib and regorafenib, additional cell-cycle arrest and also necrotic cell death. These results are supported with computational biology and also *in vivo* study. Despite this vast range of anti-HCC effect, there are still works to prove to develop new drug. First, much more strong MoA between target protein and derivatives. We showed interaction between compounds and p-JNKs with CETSA and cellular protein expression, and also activation of c-JUN and nuclear translocation but these results may have considered as an indirect evidence. CETSA showed significant thermal shift at 55°C. Though, complete CETSA melting curves across a temperature gradient or microscale thermophoresis (MST) with purified p-JNK will convince binding affinity between compounds and p-JNK. We showed strong necrotic cell death by SET135 and SET171 and described as necroptosis and ferroptosis. However, still needed more detailed experiment to prove how SET135 and SET171 induce apoptosis necrosis at the same time. Immune microenvironment is considered as a critical role in regulating tumor progression and resistance to anti-HCC compounds.^71^^,^^72^ We have needed to perform further study that how our new six derivative can show anti-HCC effect on tumor microenvironment (TME) *in vitro* and *in vivo* animal model. Major organ histology of 21 days mouse treatment with 10 to 20 mg/kg dose and GLP-like toxicity test of 7 days rat treatment with 30–300 mg/kg dose showed JNK-IN-5A derivatives are not strong toxic. However, to develop new drug pharmacokinetics analysis *in vivo* is demanded.

## Resource availability

### Lead contact

Further information and requests for resources and reagents should be directed to and will be fulfilled by the lead contact, Adil Mardinoglu (adilm@scilifelab.se).

### Materials availability

This study did not generate new unique reagents.

### Data and code availability

• RNA-sequencing data generated in this study have been deposited in the Gene Expression Omnibus and are publicly available under accession number GSE255617. This study did not generate original codes.
• Microscopy data reported in this paper will be shared by the lead contact upon request. Any additional information required to reanalyze the data reported in this paper is available from the lead contact upon request.

## Significance statement

This study identifies JNK/c-Jun pathway as a therapeutic target to hepatocellular carcinoma and demonstrates that JNK-IN-5A derivatives induce distinct apoptotic and necrotic cell death mechanisms, offering a promising therapeutic strategy beyond current kinase inhibitors.

## Acknowledgments

The authors would like to acknowledge financial support from ScandiEdge Therapeutics and the 10.13039/501100004063Knut and Alice Wallenberg Foundation.

## Author contributions

Conceptualization, W.K. and A.M.; methodology, W.K., H.J., M.O., P.M., X.L., M.L., S.I., J.S., S.A, and B.B.; writing – original draft, W.K., H.J., M.O., P.M., X.L., and M.L.; writing – review and editing, all authors involved editing and approved the final version of the article; supervision, A.M., M.U., C.Z., X.S., J.B., and H.T.

## Declaration of interests

A.M., J.B., and M.U. are the shareholders of ScandiEdge Therapeutics AB.

## STAR★Methods

### Key resources table

**Table:** 

REAGENT or RESOURCE	SOURCE	IDENTIFIER
**Antibodies**		

PARP	Cell signaling	Cat# 9542S; RRID: AB_2160739
Caspase 3	Abcam	Cat# ab32042; RRID: AB_725947
Caspase 9	Abcam	Cat# ab2324; RRID: AB_302981
Bcl-2	Abcam	Cat# ab182858; RRID: AB_2715467
STAT1	Sigma-Aldrich	Cat# HPA000982; RRID: AB_1080099
PKL	Sigma-Aldrich	Cat# HPA006653; RRID: AB_2667632
PKM	Cell signaling	Cat# 4053S; RRID: AB_1904096
JNKs	Abcam	Cat# ab179461; RRID: AB_2744672
JNK1	Abcam	Cat# ab199380; RRID: AB_3095624
JNK2	Abcam	Cat# ab76125; RRID: AB_1310369
phospho-JNKs	Abcam	Cat# ab124956; RRID: AB_10973183
c-Jun	Sigma-Aldrich	Cat# HPA059474; RRID: AB_2684030
phospho-*c*-Jun	Invitrogen	Cat# MA5-15115; RRID: AB_10979594
GAPDH	Abcam	Cat# ab8245; RRID: AB_2107448
p53	Abcam	Cat# ab32389; RRID: AB_776981
p21	Abcam	Cat# ab109199; RRID: AB_10861551
p-S349 p62	Abcam	Cat# ab211324; RRID: AB_2885114
p62	Abcam	Cat# ab109012; RRID: AB_2810880
c-Myc	Abcam	Cat# ab32072; RRID: AB_731658
GPX4	Abcam	Cat# ab125066; RRID: AB_10973901
MMP9	Invitrogen	Cat# MA5-32705; RRID: AB_2809982
β-actin	Abcam	Cat# ab8227; RRID: AB_2305186
α-tubulin	Abcam	Cat# ab7291; RRID: AB_2241126
Goat Anti-Rabbit HRP	Abcam	Cat# ab205718; RRID: AB_2819160
Goat Anti-mouse IgG-HRP	Santa Cruz Biotechology, Inc.	Cat# sc2005; RRID: AB_631736
Lamin B1	Abcam	Cat# ab16048; RRID: AB_443298
LC3A/LC3B	Invitrogen	Cat# PA1-16931; RRID: AB_2137583
ki-67	Proteintech	Cat# 27309-1-AP; RRID: AB_2756525
Cleaved-PARP	Proteintech	Cat# 60555-1-Ig; RRID: AB_3743221

**Chemicals,peptides,andrecombinantproteins**		

DMEM	Sigma-Aldrich	Catalog #: D0819
RPMI	Sigma-Aldrich	Catalog #: R2405
Fetal bovine serum	Sigma-Aldrich	Catalog #: F7524
Penicillin-Streptomycin	Sigma-Aldrich	Catalog #: P4333
JNK-IN-5A	MedChemExpress	Catalog #: HY-15881
Necrostatin-1	Santa Cruz Biotechnology	Catalog #: sc-200142
Ebselen	MedChemExpress	Catalog #: HY-13750
Sorafenib	MedChemExpress	Catalog #:HY-10201
Regorafenib	MedChemExpress	Catalog #:HY-10331
Rat tail collagen	Sigma-Aldrich	Catalog #: C3867
Propodium iodide	Sigma-Aldirich	Catalog #: P3566

**Criticalcommercialassays**		

MTT assay	ThermoFisher	Catalog #: M6494
LDH assay kit	Abcam	Catalog #: ab65393
CelLytic M	Sigma-Aldrich	Catalog #: C2978
2x Laemmli Sample Buffer	Biorad	Catalog #: 1610737
TMRE-Mitochondrial Membrane Potential Assay Kit	Abcam	Catalog #: ab113852
Autophagy Assay Kit	Abcam	Catalog #: ab139484
DCFDA/H2DCFDA - Cellular ROS Assay Kit	Abcam	Catalog #: ab113851
Lipid Peroxidation Assay Kit (Cell-based)	Abcam	Catalog #: ab243377
RNeasy Plus Mini Kit	QIAGEN	Catalog #: 74134
RNA 6000 Nano kit	Agilent	Catalog #: 5067-1511
TUNEL BrightGreen Apoptosis Detection Kit	Vazyme	Catalog #: A112-02
Caspase-3 assay kit	Abcam	Catalog #: ab39401
Universal two-step detection kit	ZSGB-BIO	Catalog #: PV-9000
enhanced Enzyme-Labeled Sheep Anti-Mouse/Rabbit IgG Polymer	ZSGB-BIO	Catalog #: PV-6000

**Depositeddata**		

RNA sequencing data of the treated HepG2 cell line	This study	GEO: GSE255617

**Experimental models: Cell lines**		

HepG2	ATCC	ATCC HB-8065ä
Huh7	iCell Bioscience Inc	iCell-h080
THLE-2	ATCC	ATCC-CRL-2706

**Experimental models: Organisms/strains**		

BALB/c nude mice (male)	Hangzhou Ziyuan Laboratory Animal Technology Co., Ltd.	Balb/c NU
Wistar rat (male)	Envigo	N/A

**Oligonucleotides**		

siPKLR origen	Origene	Catalog #:SR321329

**Software andalgorithms**		

FastQC (v0.11.9)	N/A	https://www.bioinformatics.babraham.ac.uk/projects/fastqc/
kallisto (v0.48.0)	Bray et al.^73^	https://pachterlab.github.io/kallisto/
tximport (v1.28.0)	Soneson et al.^74^	https://github.com/thelovelab/tximport
DESeq2 (v1.40.2)	Love et al.^75^	https://github.com/thelovelab/DESeq2
pcaMethods (v1.92.0)	Stacklies et al.^76^	https://github.com/hredestig/pcaMethods
msigdbr (v7.5.1)	Liberzon et al.^77^	https://github.com/igordot/msigdbr
clusterProfiler (v4.8.2)	Wu et al.^78^^,^^79^	https://github.com/YuLab-SMU/clusterProfiler
progeny (v1.24.0)	Schubert et al.^61^	https://github.com/saezlab/progeny
decoupleR (v2.8.0)	Badia-i-Mompel et al.^80^	https://github.com/saezlab/decoupleR
GOSemSim (v2.26.1)	Yu et al.^58^	https://github.com/YuLab-SMU/GOSemSim
R (v4.3.1)	N/A	https://www.r-project.org/

### Experimental model and study participant details

#### Cell culture, cell viability and cytotoxicity assay

HepG2 human liver cancer cell lines were maintained and used with RPMI 1640 (R2405, Sigma-Aldrich) supplemented with 10% fetal bovine serum (F7524, Sigma-Aldrich), 1% P/S (P4333, Sigma-Aldrich). Huh7 liver cancer cell line and THLE-2 non-tumorigenic human liver epithelial cell line were cultured using DMEM high glucose (D0819, Sigma-Aldrich) with 10% FBS and 1% P/S. 20,000 cells per well of HepG2 cells were seeded into a 96-well plate as triplicated for MTT assay and LDH assay. Cell viability was measured by MTT assay (M6494, ThermoFisher) and cytotoxicity was measured by LDH assay kit (ab65393, Abcam) by following the manufacturer’s instructions. Optical density (O.D) had been enumerated with a microplate reader (Hidex Sense Meta Plus).

### Method details

#### Western blot and CETSA analysis

For CETSA, one million HepG2 cells were treated with 20 μM compounds for 1hrs. Cells were washed and prepared into 100 μL PBS in 1.5 mL Eppendorf tube. Heat shock was given by MultiTherm Shaker (H5000-HC, Benchmark scientific) at 50°C for 3min 300rpm. Proteins were extracted with freeze thaw method using liquid nitrogen. After three times freeze and thaw, samples were centrifuged at 4°C for 20min max speed. HepG2 cells were seeded into a 6-well plate at 400,000. After two days of 10 μM small molecule treatment, lysates were harvested with CelLytic M (C2978, Sigma-Aldrich). 20 μg lysate was prepared with 2x Laemmli Sample Buffer (1610737, Biorad). SDS PAGE were performed using Mini-PROTEAN TGX Precast Gels (Bio-Rad) and transferred using Trans-Blot Turbo Transfer System (Bio-Rad). Primary antibody, PARP (95425, Cell signaling), Caspase 3 (ab32042, Abcam), Bcl-2 (ab182858), STAT1 (HPA000982, Merk), PKL (06653, Sigma), PKM (4053S, Cell signaling), JNKs (ab179461, Abcam), JNK1 (ab199380, Abcam), JNK2 (ab76125, Abcam), phospho-JNKs (ab124956, Abcam), c-Jun (HPA059474), phospho-*c*-Jun (MA6-15115, Invitrogen), GAPDH (ab8245, Abcam), p53 (ab32389, Abcam), p21 (ab109199, Abcam), p-S349 p62 (ab211324, Abcam), p62 (ab109012, Abcam), LC3A/LC3B (PA1-16931, Invitrogen), c-Myc (ab32072, Abcam), GPX4 (ab125066, Abcam), MMP9 (MA5-32705, Invitrogen), β-actin (ab8227, Abcam), Lamin B1 (ab16048, Abcam), and α-tubulin (ab7291, Abcam) were blotted for overnight and Goat Anti-Rabbit HRP (ab205718) and goat anti-mouse IgG-HRP (sc2005, Santa Cruz Biotechnology, Inc.) were blotted for secondary antibody 1 h. ImageQuantTMLAS 500 (29-0050-63, GE) has been used to detect protein bands.

#### FACs analysis

Compounds were treated to 400,000 HepG2 cells at 10 μM for 2 days in 6 well plates. Cells were fixed with 70% ethanol and stained with 50 μg/ml PI. Prepared cells were analyzed using BD FACS Canto II Flow Cytometry system at PE-A channel 10,000 cells per group. Data were histogram plotted with BD FACSDiva 9.0.

#### Mitochondria membrane potential, autophagy, ROS, and lipid peroxidation assay

HepG2 cells were seeded into 6-well plate at 400,000 cells per well. After the desired concentration and time of compound treatment, TMRE (ab113852, Abcam), Autophagy (ab139484, Abcam), ROS (ab113851, Abcam), and Lipid peroxidation (ab243377, Abcam) assay were performed by manufacturer’s instruction. The fluorescence image was taken using ZOE Fluorescent Cell Imager (Biorad) and the ROI of fluorescence intensity was measured and calculated by ImageJ. For the microplate reader fluorescence assay, 40,000 HepG2 cells were seeded into a 96-well plate as triplicated. GFP (Ex480nm, Em530nm) and RFP (Ex549nm, Em574nm) were measured with a microplate reader (Hidex Sense Meta Plus).

#### Caspase-3 activity assay

400,000 HepG2 cells were seeded into 6-well plate and compounds were treated for 2days with 10 μM. Proteins were extracted and caspase-3 activity were measure based on substrated labeled DEVD-pNA catalysis (ab39401, Abcam). We measured activity with microplate reader (Hidex Sense Meta Plus) at O.D 400 nm.

#### Invasion assay and wound healing assay

The effects of the drugs on the invasion ability of HepG2 cells were determined by an 8 μm pores transwell chamber (Corning 3464, USA). The transwell membrane was coated with 100 μL collagen (Sigma C3867, USA) that was diluted with PBS at 1 mg/ml concentration. After overnight incubation at 4°C, discard the collagen solution and washed with PBS. 100,000 cells in 200 μL serum-free media with drugs were added into the upper well and 600 μL of 10% FBS-supplemented growth media were added to the lower well as an invasive attractant in 24 well plates. Cells were incubated in a 37°C CO_2_ incubator for one day. Cells were fixed with 3.7% formaldehyde for 30 min at room temperature and washed with PBS twice. Cells were stained with 1% crystal violet in PBS for 20 min and residual cells in the inner membrane were swabbed gently with a cotton swab. Invaded cells located outer membrane were imaged by ZOE Fluorescent Cell Imager (Bio-Rad, USA) and counted by image. For the wound healing assay, 100,000 cells were seeded into 24-well plates for each well and grown until 95–100% confluent. The linear wound was created by using a 200 μL pipette tip. Image was recorded at the time point of day 0 until control group wound closure with 1 μM drugs by ZOE Fluorescent Cell Imager (Bio-Rad, USA). Wound areas were measured using ImageJ software.

#### RNA extraction and sample preparation for RNA sequencing

RNA samples for RNA sequencing were extracted with RNeasy Plus Mini Kit (74134, QIAGEN). RIN score of total RNA samples was measured using RNA 6000 Nano kit (5067-1511, Agilent). 2100 Bioanalyzer Instrument (G2939BA, Agilent) was used for RIN score test using electrophoresis. Samples with RIN score more than 8 were submitted to SZA Omics, Istanbul for RNA sequencing analysis.

#### *In vivo* anti-tumor activity evaluation

All animal experiments were conducted in accordance with the guidelines and approved by the Welfare Ethics Committee of Zhengzhou University, Laboratory Animal Center (Approval number: ZZU-LAC20240322[01]). Male BALB/c nude mice (aged 5 weeks) were purchased from Hangzhou Ziyuan Laboratory Animal Technology Co., Ltd. (Zhejiang, China). To establish hepatocellular carcinoma xenograft tumor models, 0.2 mL serum-free cell culture medium containing 1×10^7^ Huh7 cells were injected into the subcutaneous area near the right scapular region of mice. Once the tumor volume reached 100 mm3, the mice were randomly divided into 7 groups and each group of mice was intraperitoneal administered daily with corresponding compound solutions including SET171(10 mg/kg, 20 mg/kg), SET135 (5 mg/kg,10 mg/kg), JNK-5A (10 mg/kg), Sorafenib (10 mg/kg) and equal volume of solvent control (45% saline, 40%PEG300, 5%Tween-80, and 10%DMSO). The tumor weight and body weight were measured every two days. After 21 days, the mice were euthanized, and the tumors were isolated and weighed. The blood and major organs were collected for pathological examinations.

#### Immunohistochemistry (IHC) experiment

The expression of Ki-67 and Cleaved-PARP in tumor tissues was detected by IHC assay according to the manufacturer' instructions (PV-9000, ZSGB-BIO). Briefly, 4 μm paraffin sections were dewaxed in xylene, dehydrated in gradient ethanol and boiled in sodium citrate antigen retrieval solution for 20 min. After cooling to room temperature, the slide was incubated with endogenous peroxidase blockers for 20 min at room temperature and treated with primary antibodies Ki-67 (27309-1-AP, Proteintech) and Cleaved-PARP (60555-1-Ig, 1:400, Proteintech, China) at 4 °C overnight. On the following day, the sections were incubated with reaction enhancement solution for 60 min and enhanced Enzyme-Labeled Sheep Anti-Mouse/Rabbit IgG Polymer (PV-6000, ZSGB-BIO) for 60 min at 37°C. Finally, these sections were stained with 3,3-diaminobenzidine (ZLI-9017, ZSGB-BIO) for 8 min and the nuclear was counterstained with hematoxylin.

#### TUNEL staining

The apoptotic cells in tumor tissues were detected using the TUNEL BrightGreen Apoptosis Detection Kit (Vazyme A112-02, Jiangsu, China) following the manufacturer’s instructions. Briefly, after dewaxing and rehydration, each slide was incubated with 100 μL of the diluted Proteinase K solution at room temperature for 20 min. Then the slices were washed with PBS for 3 times, and immersed with Equilibration Buffer and TdT incubation buffer. Finally, the nuclear were stained using fresh DAPI solution at room temperature for 5 min, and with Mounting Medium, antifading (Solarbio S2100, China). All slides were observed using the 3DHISTECH/Pannoramic MIDI Ⅱ microscope. ImageJ software was used to count the number of positive cells.

#### Pathological examinations

The histomorphology of the organ tissues was examined by Hematoxylin and Eosin (H&E) staining. Briefly, major organs including heart, liver, spleen, lung, kidney and tumor tissues were fixed in 4% paraformaldehyde for 48 h, then dehydrated with gradient ethanol and xylene, and embedded in paraffin. 4 μm thick paraffin sections were obtained with a microtome, deparaffinized and then subjected to H&E staining on a CW-100 tissue stainer (Ningbo Chiwell Biotechnology Co., Ltd, Zhejiang, China) as recommended by the manufacturer of the dye (Zhuhai Baso Biotech Co., Guangzhou, China).

#### Haematological tests

Haematological tests include routine blood tests and blood biochemistry tests. About 50 μL of fresh mouse blood conducted routine blood tests was firstly treated with EDTA-2K anticoagulation, and the complete blood cell counts were obtained using a veterinary haematology analyser (Hemavet950FS, Drew Scientific, Waterbury, CT, USA). For blood biochemistry tests, approximately 300 μL of fresh blood was collected in 1.5 mL centrifuge tube (without anticoagulant or preservative), allowed to stand for 30min at room temperature, and centrifuged at a relative centrifugal force (RCF) of 14,000 g for 10min. The upper layer of serum was then transferred to a clean centrifuge and tested on an automatic biochemical analyser (Indiko, Thermo, Massachusetts, USA) using commercial kits (Zhongtuo Biological Co., Shandong, China) for liver and kidney function testing.

### Quantification and statistical analysis

#### RNA-sequencing

The quality, quantity, and RNA integrity number (RIN) of the samples were assessed with NanoDrop (Thermo Fisher, USA), and Bioanalyzer (2100, Agilent Technology, USA). For the RNA samples, the ratio of absorbance at 260 and 280 nm (A260/280) of ∼2, A260/230 of 2.0–2.2, and RIN >8.0 are acceptable. All samples passed the quality control.

The Illumina Stranded Total RNA Prep, Ligation with Ribo-Zero Plus kit was used for the construction of NGS libraries from the samples with acceptable quality parameters. In short, RNA samples were sequenced with 2x100 paired-end reads by the NovaSeq 6000 system. Raw sequencing data (.bcl) were demultiplexed and converted to FASTQ files with DRAGEN Software (v3.9.5). The data was delivered in FASTQ format using Illumina 1.8 quality scores.

#### RNA-seq data pre-processing

The quality of FASTQ files was assessed by FastQC (v0.11.9) software to ensure the quality of every sequenced sample. Kallisto (v0.48.0)^73^ was used to quantify the count and transcripts per million (TPM) values of transcripts based on the Homo sapiens reference cDNA (version GRCh38, Ensembl release 110). The transcript read counts and TPM values were assembled to gene level using the R package tximport (v1.28.0),^74^ with only protein-coding transcripts and genes included. The lowly expressed genes with an average count below 5 were filtered out, resulting in a total of 14,592 genes for the downstream analysis.

#### Differential expression analysis

Differential expression analysis was conducted using the R package DESeq2 (v1.40.2)^75^ followed by the Benjamini-Hochberg procedure to correct *p*-values. Adjusted *p*-value <0.05 was chosen as the threshold for the significance of differentially expressed genes (DEGs), with log2 fold change >1 for up-regulation and log2 fold change < −1 for down-regulation.

#### GLP-like toxicity study in rats

A 7-day oral toxicity study was conducted in male Wistar rats (supplier: Envigo, Venray, Netherlands) to preliminarily assess the tolerability of SET135 and SET171 at dose levels of 30, 100, and 300 mg/kg body weight. The study included a vehicle control group (4 males and 4 females) and three dose groups for each compound, with each dose group consisting of 3 male and 3 female rats. The vehicle comprised 1.5% (w/w) hydroxypropyl methylcellulose (HPMC) and 1.5% (w/w) polysorbate 80 (PS80) in 10 mM phosphate-buffered saline (PBS, pH 7), administered at a dosing volume of 5 mL/kg. Dosing was performed once daily in the morning for seven consecutive days. For hematology, 0.5 mL of blood was collected into K_2_EDTA tubes per animal; for plasma chemistry, 0.6 mL was collected into lithium heparin tubes. All samples were analyzed within 60 min of collection. The Exigo hematology analyzer was calibrated against a reference sample provided by the manufacturer, and a full control sample analysis cycle was performed prior to analysis. All the animal procedures and ethical review were performed according to the 2010/63/EU Directive on the protection of animals used for biomedical research.

#### Principal component analysis

Principal component analysis (PCA) was applied to visualize the distribution of the samples, which was performed by the R package pcaMethods (v1.92.0)^76^ based on the gene expression profiles after variance stabilizing transformation by DESeq2.

#### Gene set analysis

Gene set overrepresentation analysis (GSOA) was applied to determine whether a list of DEGs of interest was significantly associated with specific Gene Ontology (GO) biological process terms in the RNA-seq dataset, with all 14,592 analyzed genes as the background. In addition, we performed gene set enrichment analysis (GSEA)^59^ to assess the activation or inhibition of biological pathways in response to the treatments. Based on this method, genes were sorted based on the log2 fold change in descending order, and the 50 hallmark gene sets from the Molecular Signatures Database (MSigDB)^77^ were tested for their significance. Here, a positive normalized enrichment score (NES) indicates that a hallmark gene set is activated in the corresponding gene list ranked by log2 fold change, and vice versa. The hallmark gene sets were retrieved from the R package msigdbr (v7.5.1).^77^ GSEA was also performed based on the KEGG pathways^60^ in the same manner. The R package clusterProfiler (v4.8.2)^78^^,^^79^ was used for both GSOA and GSEA, with *p*-values adjusted by the Benjamini-Hochberg procedure. An adjusted *p*-value <0.05 was considered significant.

In addition, the PROGENy analysis^61^ was performed to infer the activity scores of the 14 cancer-related pathways. For this, the log2 fold change values of each treatment vs. control were used as input, and the relative scores across all nine treatments were inferred using the R package progeny (v1.24.0) and decoupleR (v2.8.0).^80^

#### Comparison of DEGs

We used three approaches to estimate the similarity between DEGs under different comparisons. First, we calculated the Jaccard index (in other words, Jaccard similarity) defined as the ratio of the size of the intersection relative to the size of the union of the two sets of DEGs. Second, we calculated the *p*-value for the overlap between DEGs by hypergeometric testing. The *p*-values were then corrected by the Benjamini-Hochberg procedure. Lastly, we used the R package GOSemSim (v2.26.1)^58^ to evaluate the semantic similarity between the significant GO biological process terms that are associated with different sets of DEGs.

#### Mechanism of action (MoA) prediction

We used our previously developed computational pipeline Open MoA^21^ for predicting the potential MoAs of the sorafenib, regorafenib, JNK-IN-5A and six derivatives. The original integrated network (IN) provided by Open MoA study was used as the reference network. Among all treatment groups and control groups, only genes with TPM value maxima greater than 1.00 were kept and matched to the IN for constructing a study-specific IN. Next, for each drug, significant DEGs with false discovery rate (FDR) smaller than 0.05 are filtered. Since JNK-IN-5A and six derivatives are novel drugs which not included in the DrugBank database and the original IN, their direct downstream target MAPK8 and MAPK9 were chosen as the starting protein. The drug-specific subnetworks were generated by setting MAPK8 or MAPK9, Sorafenib, and Regorafenib as the starting points and their significant DEGs as the endpoints respectively, followed by the confidence score calculation for edge weights in each subnetwork via Open MoA. The most potential MoA for each drug was predicted through the shortest paths analysis from drugs, MAPK8, or MAPK9 (starting points) to genes TP53 or MYC (endpoints) by Open MoA pipeline. All procedures including drug-specific subnetwork construction, confidence scores calculation, and shortest path analysis are performed according to the Open MoA pipeline by R (v4.3.1).

#### Statistical analysis for RNA-seq and experimental data

Error bars shown in the graphs are presented as the mean ± standard deviation (SD). For RNA-seq data analysis, *p*-values were adjusted using the Benjamini-Hochberg procedure where applicable. Statistical significance was defined as *p*-value <0.05 (or adjusted *p*-value <0.05), indicated by an asterisk (∗).

## Supplemental information

Document S1. Figures S1–S7

Data S1. Raw image for western blots.pdf

Data S2. STR report HepG2.pdf

Data S3. STR report Huh7.pdf
