# Supplementary material for: Phospho-JNK agonists show promising effects for the treatment of hepatocellular carcinoma
Source: iScience. 2026 May 20;29(6):116005. doi: 10.1016/j.isci.2026.116005 (PMC13214269; doi:10.1016/j.isci.2026.116005)
Supplement: Document S1. Figures S1–S7 [file mmc1.pdf]

iScience, Volume *29*

Supplemental information

Phospho-JNK agonists show promising effects for the treatment of hepatocellular carcinoma

Woonghee Kim, Han Jin, Peipei Miao, Mehmet Ozcan, Xinmeng Liao, Mengzhen Li, Shazia Iqbal, Jihad Sebhaoui, Sajda Ashraf, Burcu Belmen, Hasan Turkez, Jan Boren, Mathias Uhlen, Xiaojing Shi, Cheng Zhang, and Adil Mardinoglu

Supplementary Figures

A

MTT assay for 1week steatosis with compounds

Compound	Cell viability (%)	SD±
Control	100	5.02
SET159	2.33	0.84
SET158	9.59	1.96
SET171	22	1.18
SET156	26.64	3.83
SET157	36.47	6.01
SET119	40.12	1.57
SET132	40.31	2.91
SET117	40.59	1.58
SET153	42.5	0.43
SET152	43.87	1.42
SET161	44.06	0.93
SET131	44.75	2.57
SET154	45.95	1.38
SET164	46.01	2.47
SET163	46.64	1.44
SET162	49.2	2.63
SET114	49.35	1.28
SET149	50.88	1.66
SET172	53.7	3.04
SET160	56.41	6.36
JNK-IN-5A	56.44	2.85
SET56	56.98	1.47
SET192	60.1	2.44
SET151	61.6	5
SET193	62.32	2.39
SET135	63.24	0.68
SET130	64.1	4.88
SET170	66.03	1.06
SET179	66.54	0.97
SET133	69.29	4.79
SET173	70.83	1.4
SET129	71.23	3.01
SET183	72.28	3
SET153	74	1.7
SET164	74.13	2.45
SET190	76.16	5.12
SET118	91	3.2

MTT assay for 2days compounds treatment

Compound	Cell viability (%)	SD±
Control	100	9.7
SET135	29.39541	0.5
SET163	31.4107	2.4
SET151	34.2599	3.8
SET149	35.37179	1.5
SET162	38.22099	2.6
SET172	38.29048	5.8
SET160	40.37526	5.4
SET114	40.51425	1.7
SET173	40.72272	0.4
SET133	41.20917	3.2
SET159	42.59903	2.9
SET56	44.40584	3.0
SET158	44.7533	5.5
SET164	45.17026	1.6
SET130	45.7262	4.3
SET170	45.7262	0.8
SET154	46.14315	2.6
SET118	46.76859	2.3
SET153	46.83808	4.9
SET193	46.83808	2.0
SET171	47.67199	1.0
SET153	51.4246	0.0
SET156	51.63308	4.6
SET161	51.77206	1.7
SET192	51.91105	4.8
JNK-IN-5A	52.60598	3.2
SET190	53.16192	3.4
SET164	55.03822	0.4
SET179	55.31619	0.0
SET129	55.52467	8.2
SET157	55.59416	0.4
SET152	56.42808	1.4
SET183	59.27728	8.0

MTT assay for 4days compounds treatment

Compound	Cell viability (%)	SD±
Control	100.0	3.0
SET171	15.2	1.5
SET135	27.4	0.1
SET149	30.9	0.2
SET114	31.4	1.0
SET172	32.7	1.5
SET162	38.0	0.4
SET130	38.3	0.3
SET158	39.5	1.2
SET154	39.6	1.4
SET193	39.9	1.6
SET153	40.4	0.8
SET159	40.8	2.6
SET133	41.3	5.6
SET170	42.3	1.2
SET173	42.4	2.4
SET163	42.6	3.3
SET161	44.5	1.8
SET157	45.1	1.8
SET192	45.6	8.9
SET156	47.0	0.5
SET151	47.8	0.9
SET153	48.1	2.3
SET160	48.2	0.8
SET190	48.7	7.5
SET152	49.6	5.7
SET118	50.5	1.0
SET164	51.0	2.0
SET164	55.3	5.6
SET129	57.0	1.7
SET183	57.2	2.5
JNK-IN-5A	61.0	3.1
SET56	63.8	13.0
SET179	72.0	4.9

B

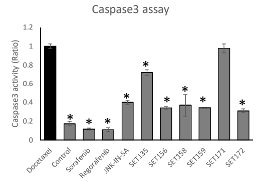

C

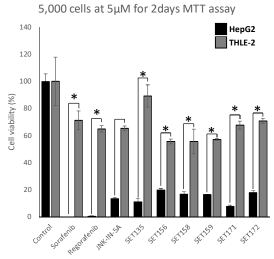

D

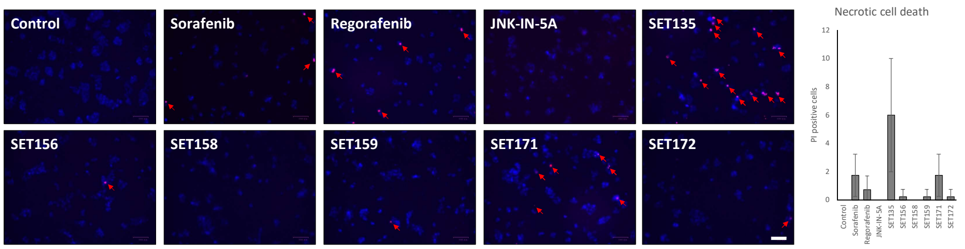

E

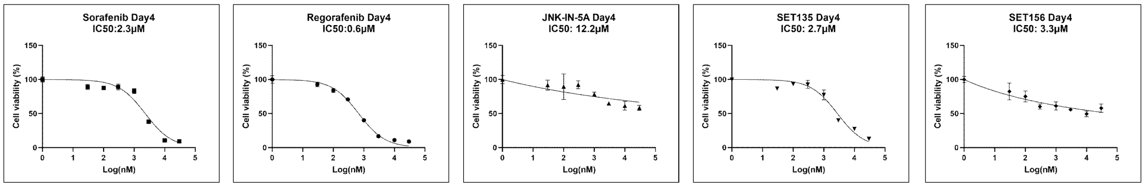

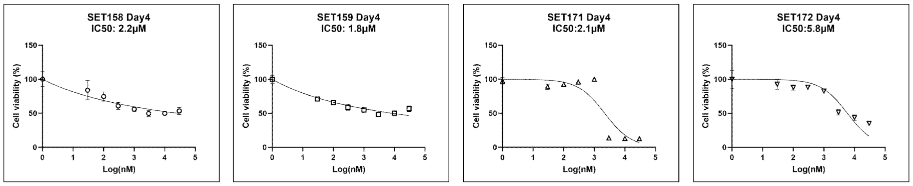

HepG2	IC50 (μM)
Sorafenib	2.3
Regorafenib	0.6
JNK-IN-5A	12.2
SET135	2.7
SET156	3.3
SET158	2.2
SET159	1.8
SET171	2.1
SET172	5.8

Supplementary Figure 1. Cell viability and cytotoxicity assay for drug candidates

(A) Cell viability assay (MTT) for JNK-IN-5A and six derivatives for one-week steatosis, four days and two days treatment at 10μM.

(B) Caspase-3 activity assay.

(C) Cell viability assay (MTT) to compare toxicity between HepG2 HCC cell line and THLE-2 non-tumorigenic human liver epithelial cell line.

(D) DAPI and PI staining for necrotic cell death analysis. Drugs were treated for 2days at 10μM and cells were stained with DAPI (Blue) and PI (Red) for 30min. at live cells. PI positive necrotic cells were counted and plotted. Scale bar = 100μm.

(E) IC_{50} value for hit compounds at day 2. 8 group of 30μM, 10μM, 3μM, 1μM, 300nM, 100nM, 30nM and untreated were observed.

Data are represented as mean +/- SD. Significance was tested using t-test (* $P < 0.05$).

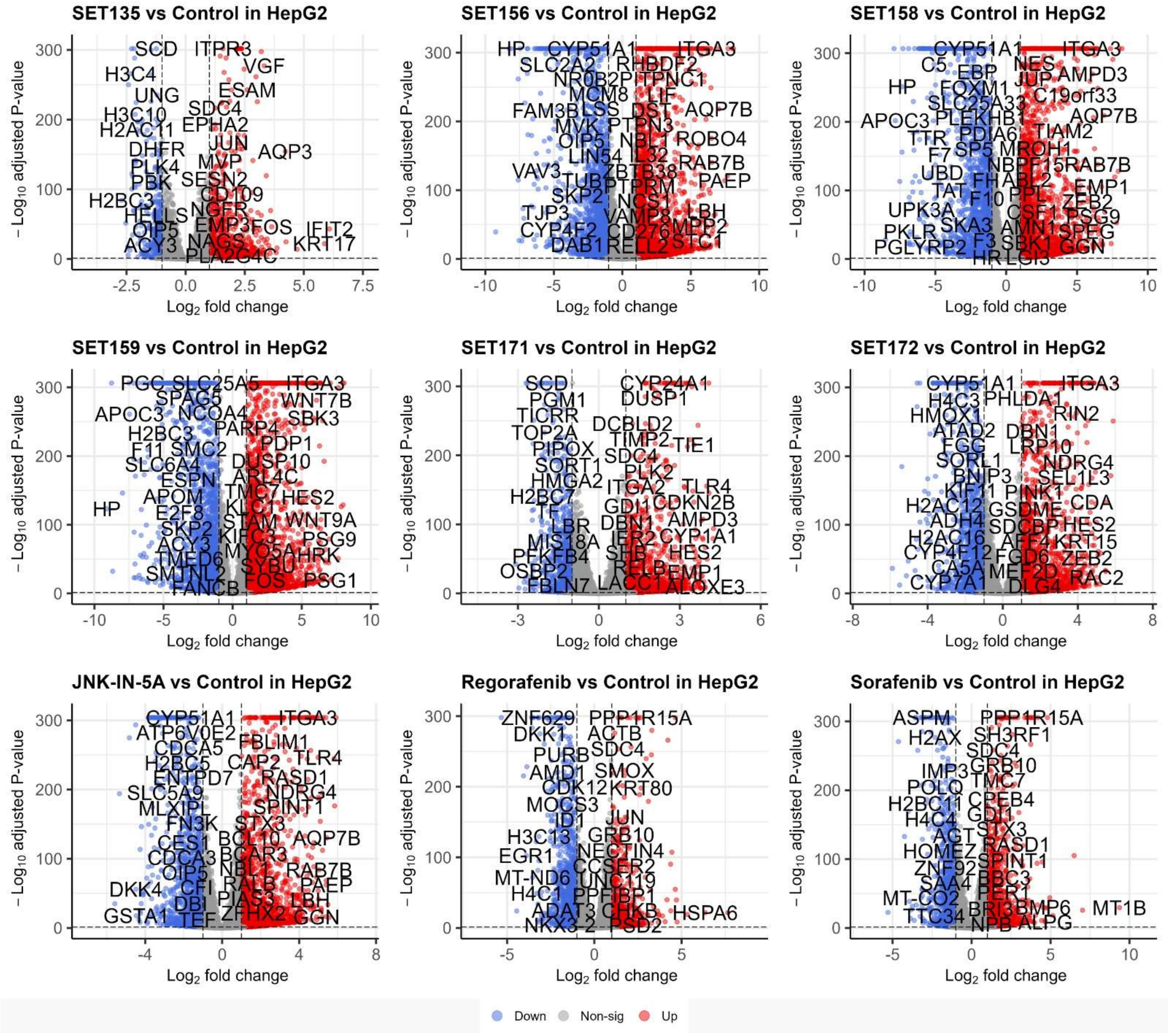

Supplementary Figure 2. Volcano plots show differentially expressed genes for each treatment vs. control in HepG2 cells.

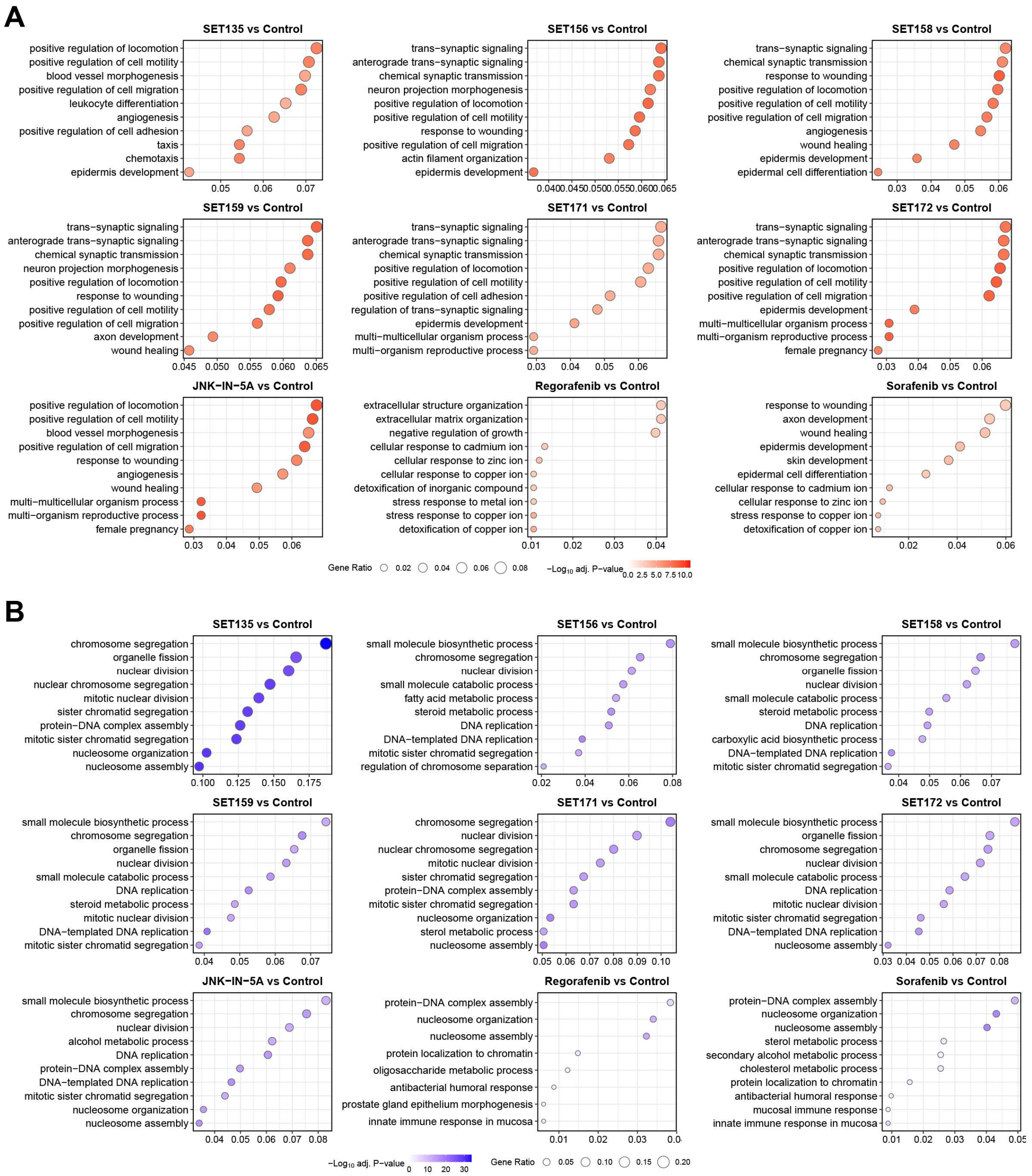

Supplementary Figure 3. GSOA based on significantly up-regulated genes (A) and down-regulated genes (B) for each treatment vs. control in HepG2 cells.

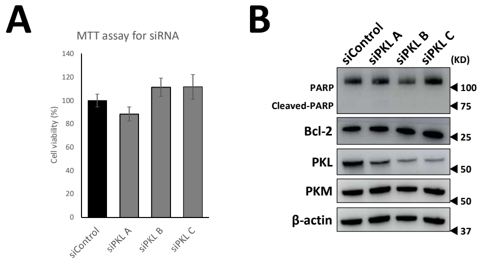

Supplementary Figure 4. PKL silencing with siRNA not induce cytotoxicity.

(A) Cell viability assay (MTT) for siRNA silenced PKL on HepG2 cells in triplicate.

(B) Western blot analysis for siRNA silenced PKL on HepG2 cells.

Data are represented as mean +/- SD.

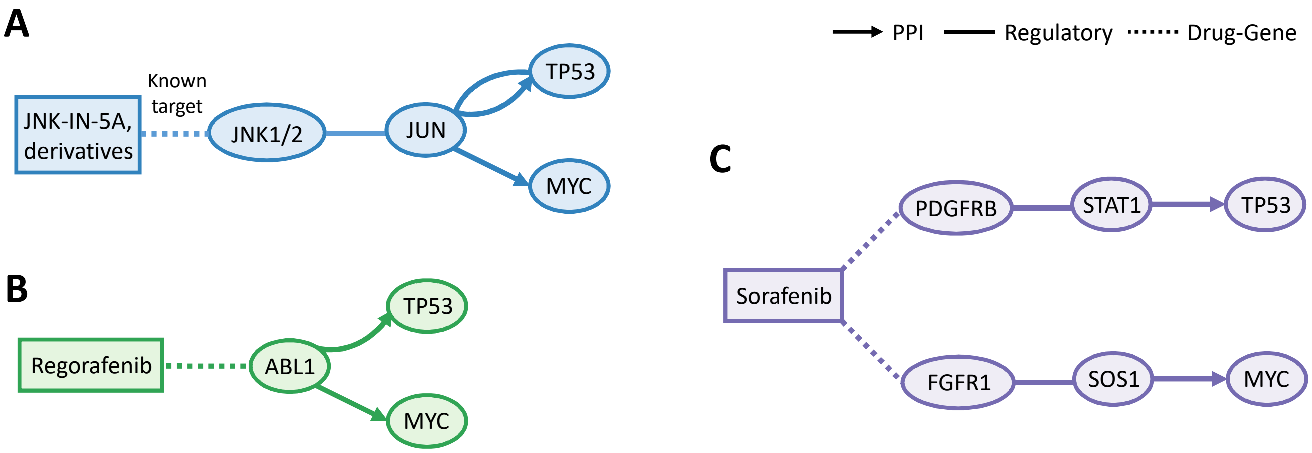

Supplementary Figure 5. Predicted MoAs by Open MoA

Prediction of the most potential mechanism of action (the shortest paths) from JNK drugs (A), Regorafenib (B), and Sorafenib (C) to protein TP53 or MYC. Notably, the six PKLR-modulating compounds and JNK-IN-5A have the same predicted pathways. Confidence scores calculated by Open MoA for all edges in the predicted MoA are greater than 0.99.

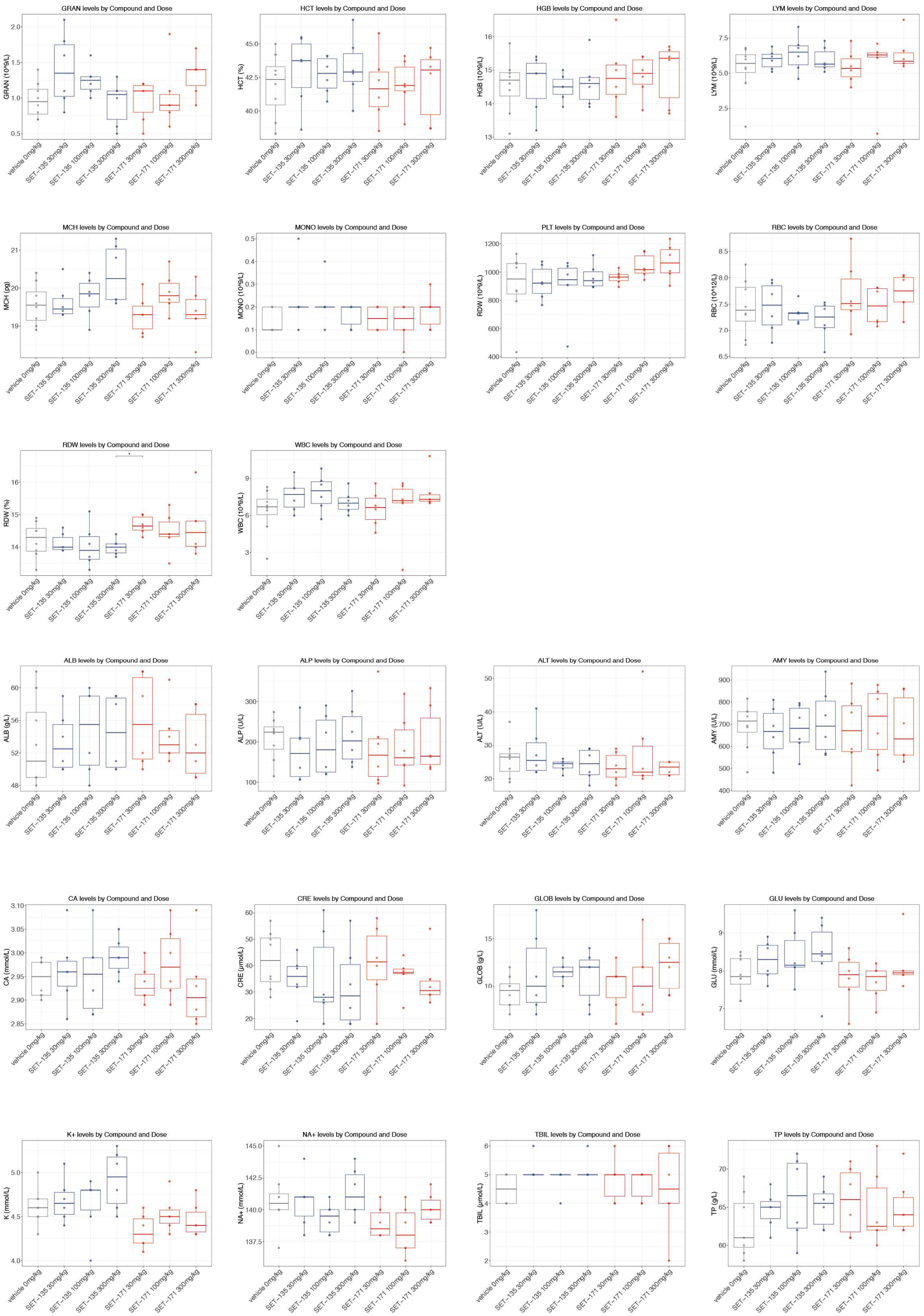

Supplementary Figure 6. Hematology and Plasma Chemistry

Hematology and plasma chemistry parameters remained within normal ranges, supporting the overall safety profile of the compound.

Data are represented as mean +/-IQR.

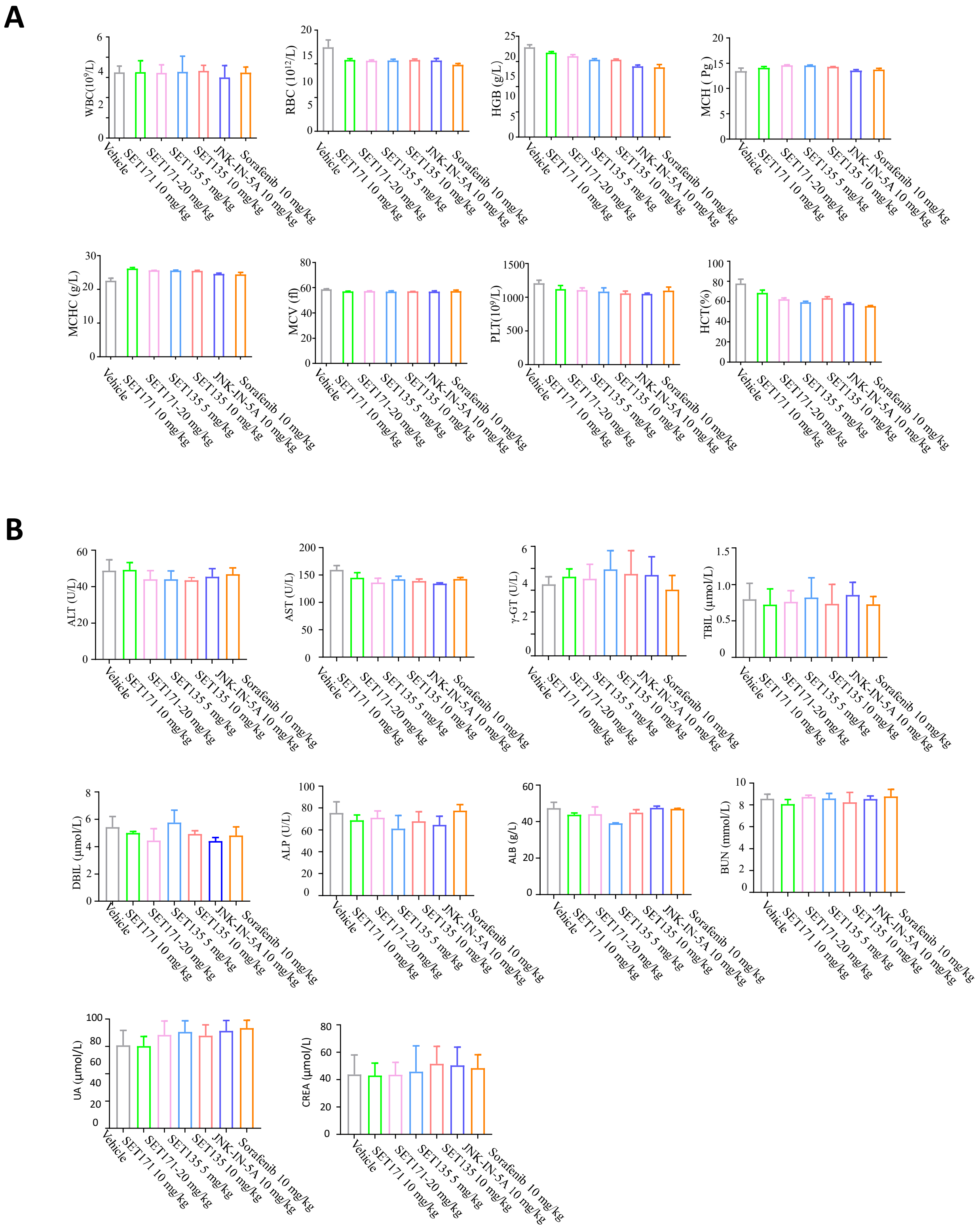

Supplementary Figure 7. SET135 and SET171 shows higher security in long-term use.
(A) Hematological test (n = 5 biologically independent animals for each group) and.
(B) serum biochemicals tests (n = 4 biologically independent animals for each group) of the mice after the daily administration of JNK inhibitors for 21 days.
Data are represented as mean +/- SD. WBC: white blood cells, RBC: red blood cells, HGB: hemoglobin, MCH: mean corpuscular hemoglobin, MCHC: mean corpuscular hemoglobin concentration, MCV: mean cell volume, PLT: blood platelet, HCT: hematocrit, ALT: alanine transferase, AST: aspartate transferase, γ-GT: γ-glutamyl transpeptidase, TBIL: total bilirubin, DBIL: direct bilirubin, ALP: alkaline phosphatase, ALB: albumin, BUN: blood urea nitrogen, UA: uric acid, CREA: creatinine.
