# Supplementary material for: Phospho-JNK agonists show promising effects for the treatment of hepatocellular carcinoma
Source: iScience. 2026 May 20;29(6):116005. doi: 10.1016/j.isci.2026.116005 (PMC13214269; doi:10.1016/j.isci.2026.116005)

Raw image for western blot

Figure1 D

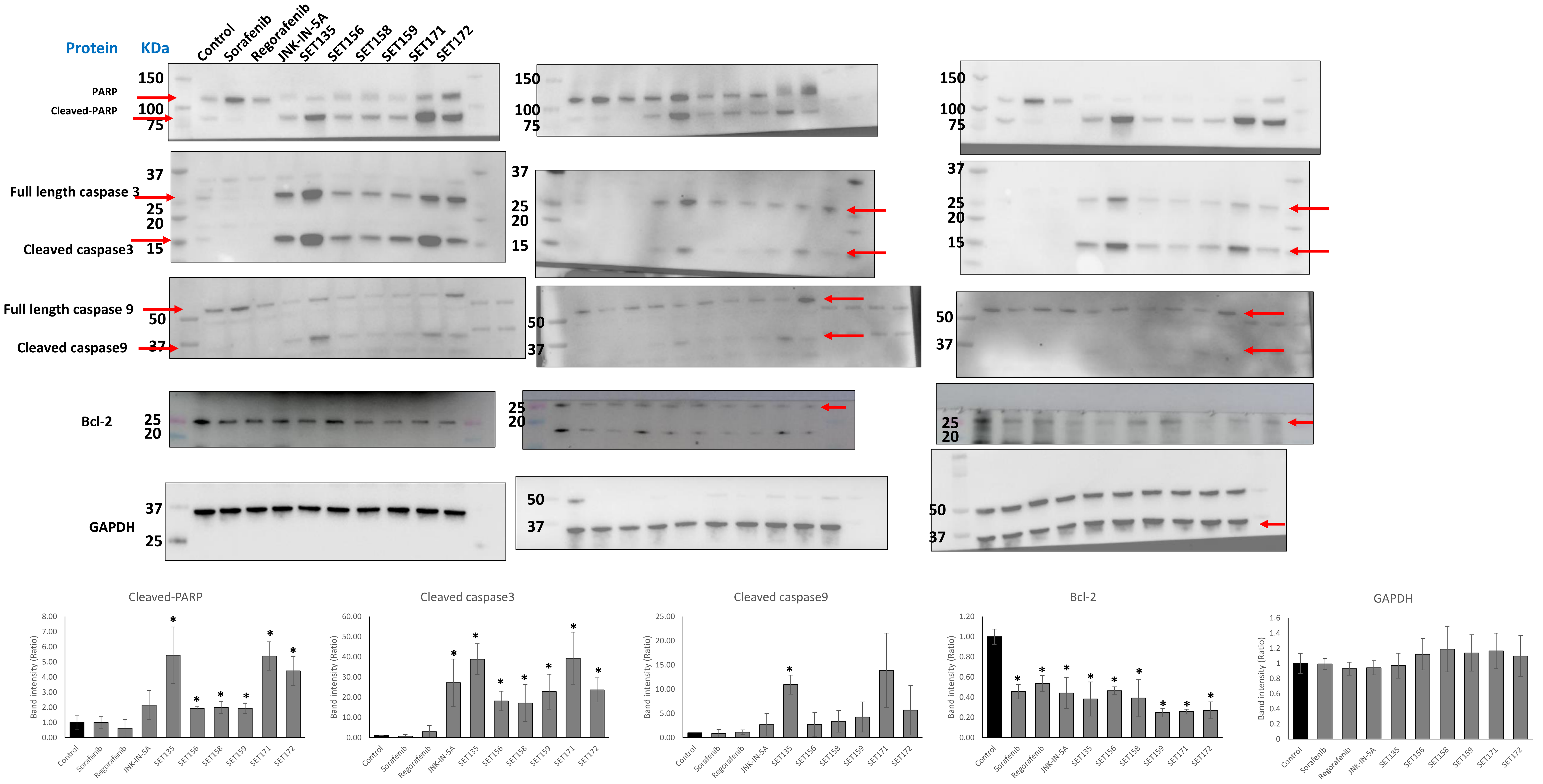

Figure1 E

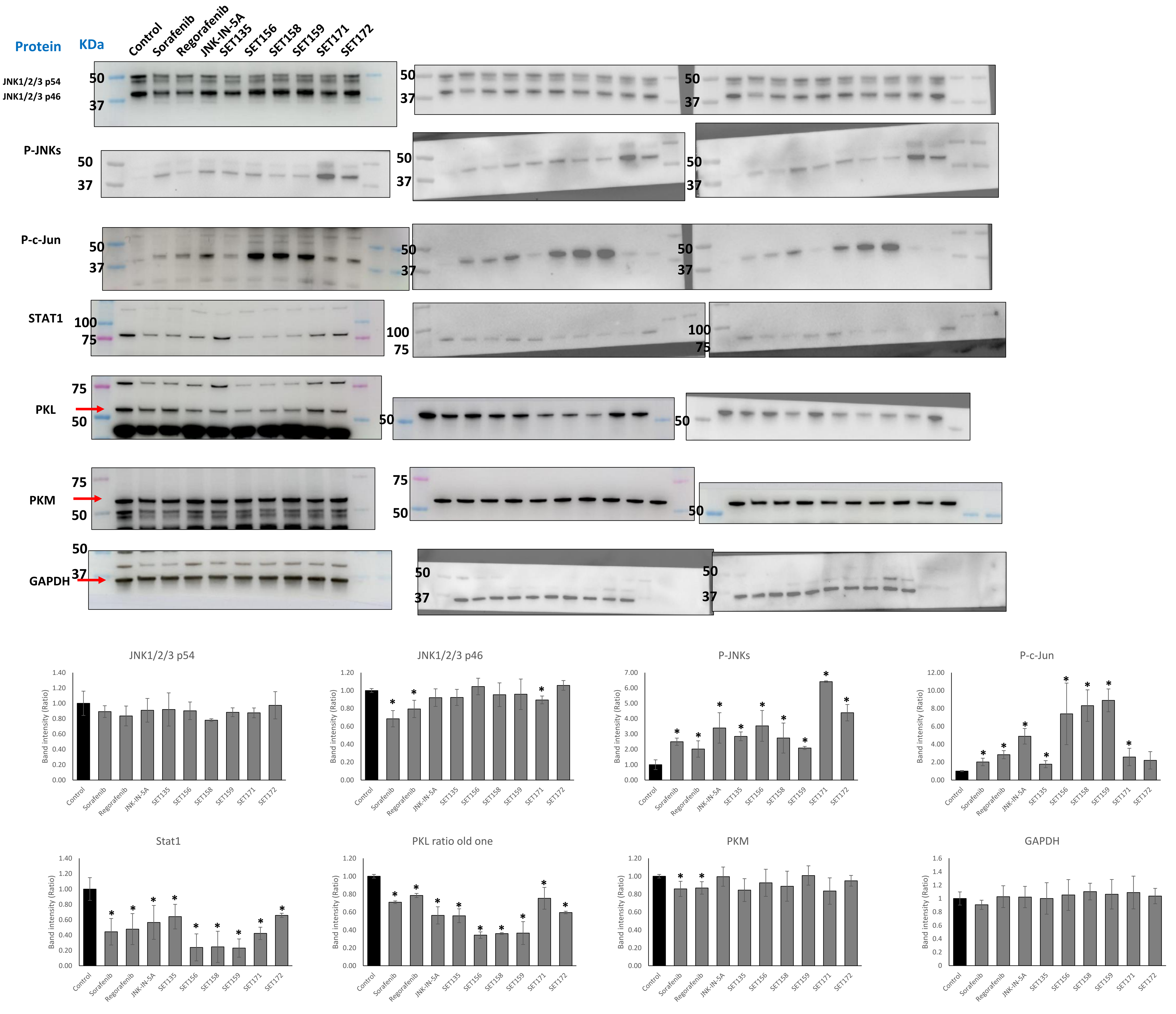

Raw image for western blot

Figure1 F

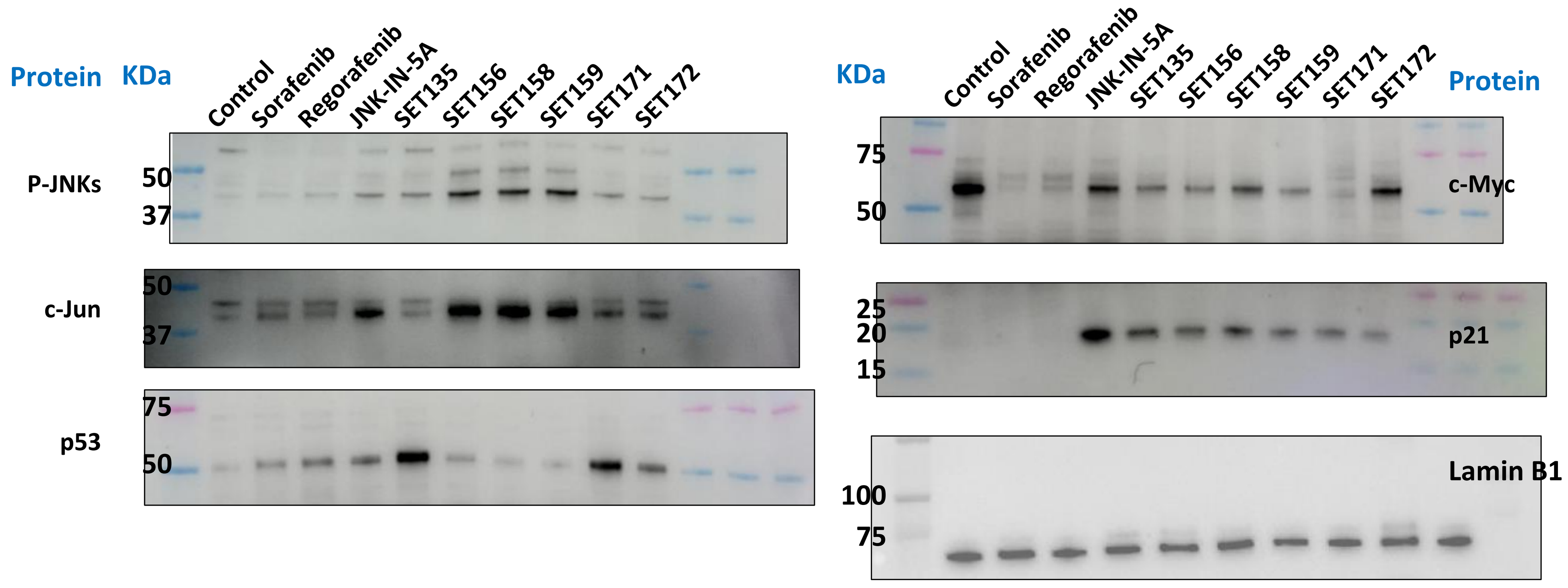

Figure1 G

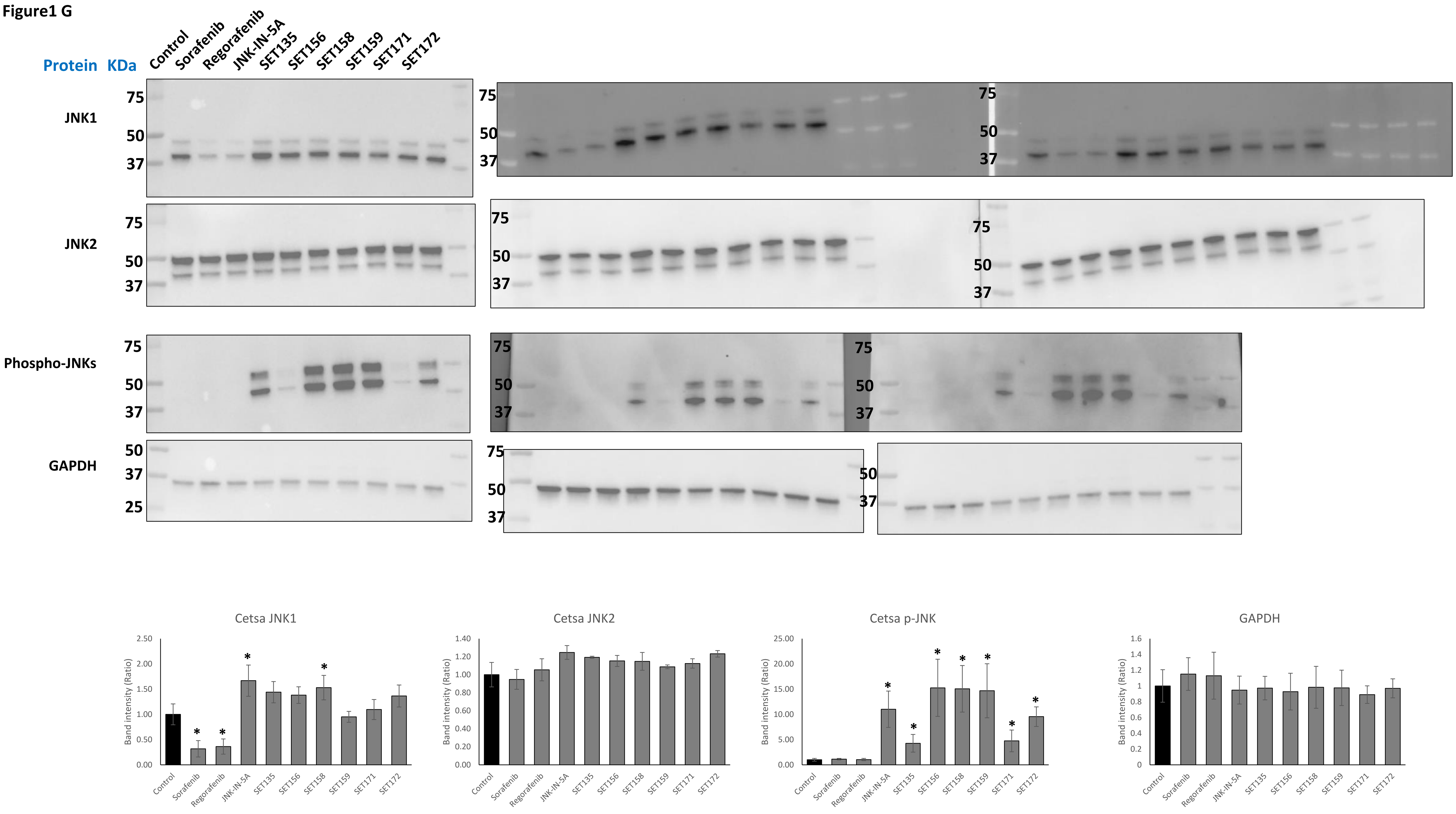

Raw image for western blot

Figure2 B

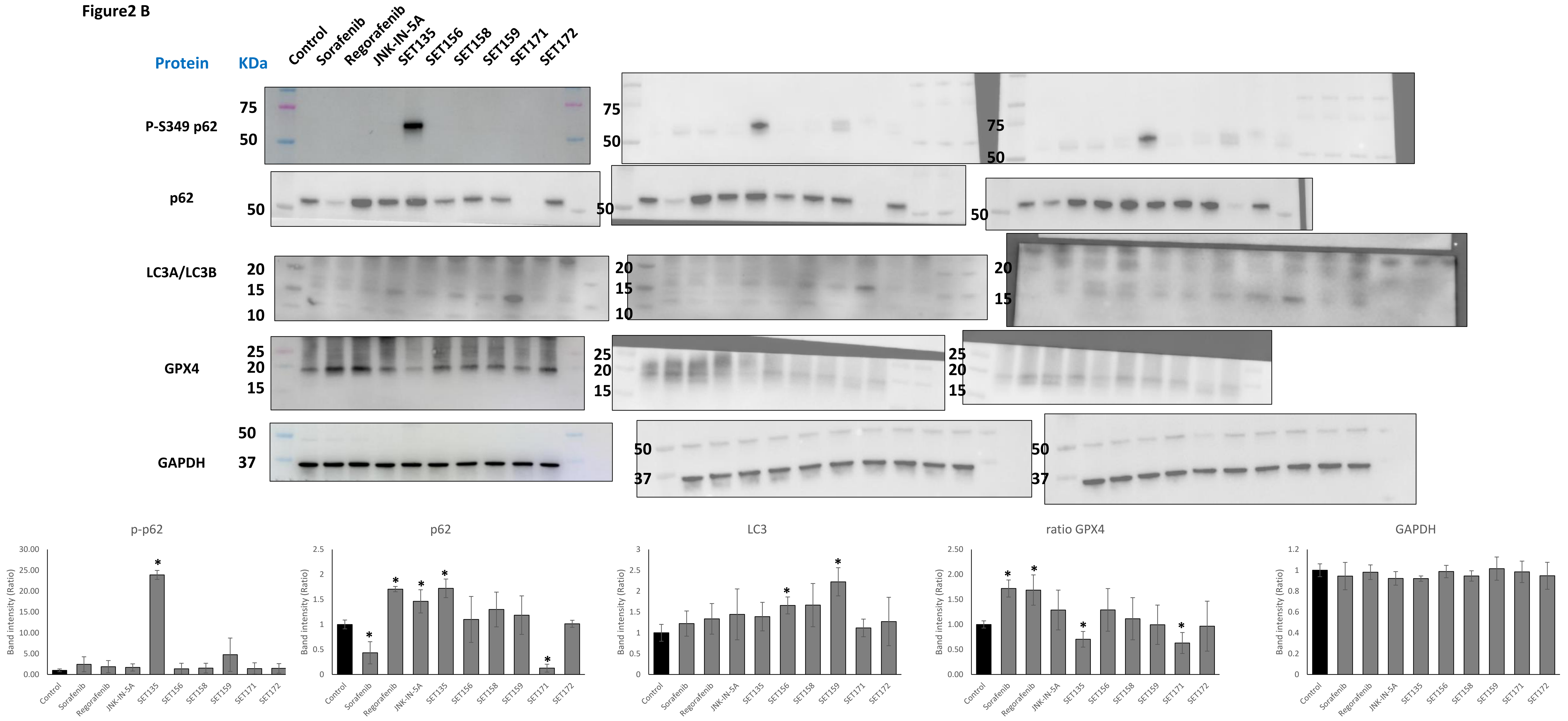

Figure3 B

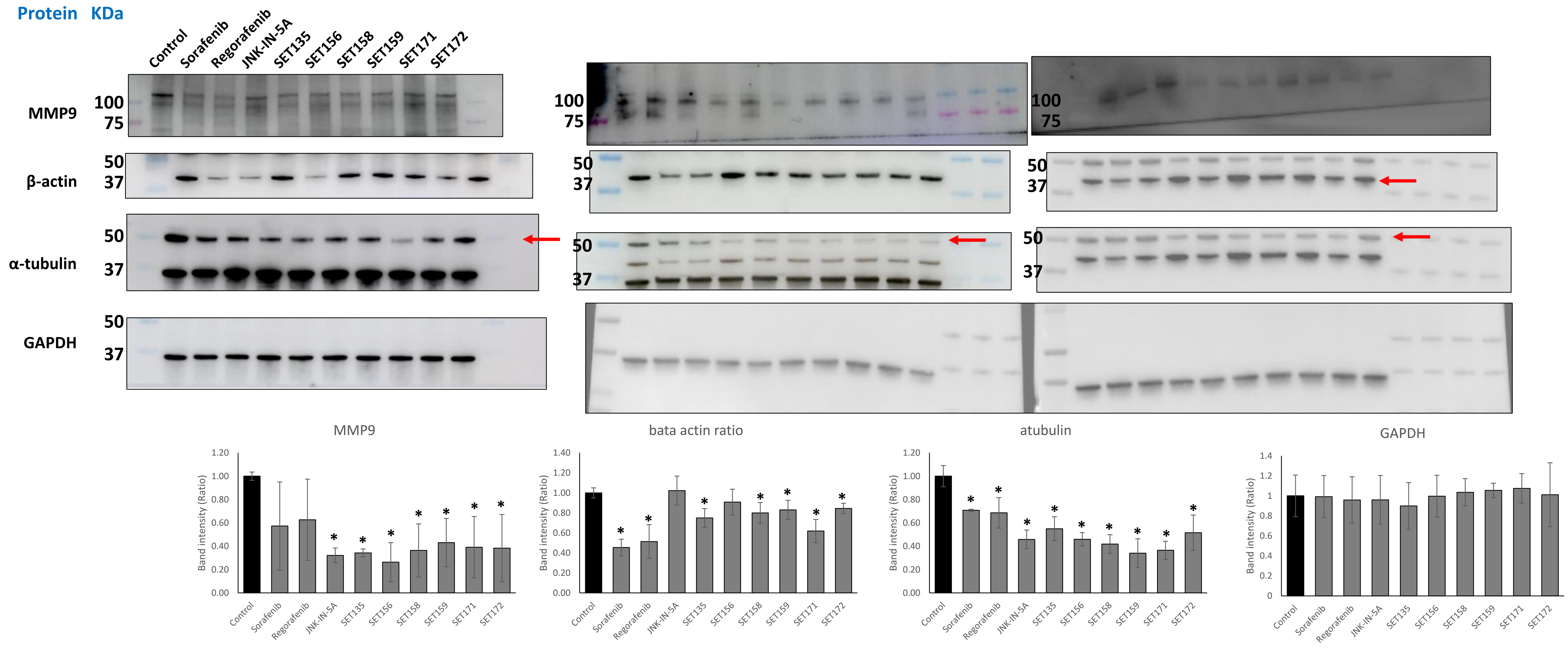

Supplementary Figure 4 B

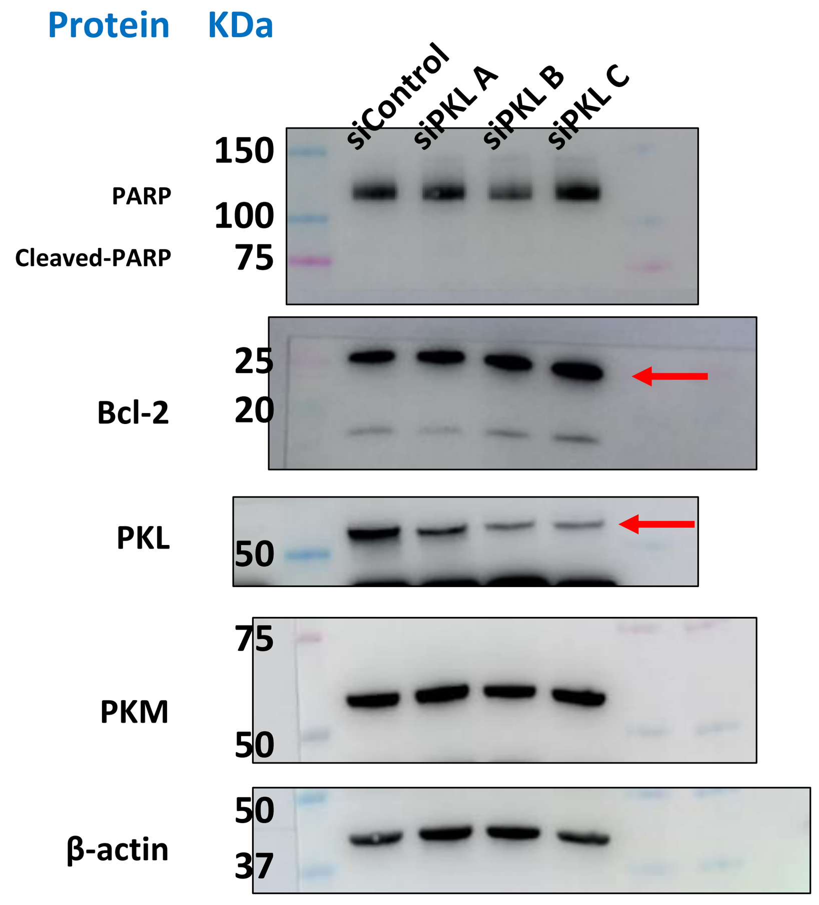

Supplement: Data S1. Raw image for western blots.pdf [file mmc2.pdf]
