# Supplementary material for: Phospho-JNK agonists show promising effects for the treatment of hepatocellular carcinoma
Source: iScience. 2026 May 20;29(6):116005. doi: 10.1016/j.isci.2026.116005 (PMC13214269; doi:10.1016/j.isci.2026.116005)
Supplement: Data S2. STR report HepG2.pdf [file mmc3.pdf]

ANALYSIS REPORT
Cell Line Authentication

Cytion

**Customer**	Scilifelab
**Sample**	HepG2
**Order Number**	AB253236
**Date**	07.11.2025

1. Analysis Method

Profiling of the cell line was done using highly polymorphic short tandem repeat loci (STRs). STR loci were amplified using the PowerPlex® 16 HS System (Promega). Fragment analysis was done on an ABI3730xl (Life Technologies) and the resulting data were analyzed with GeneMarker HID software (Softgenetics).

2. Results

2.1. STR-Profile

Locus	*Chromosomal Location*	*Core STR Marker*	*Customer Sample Typed Alleles*	*Database Alleles*
D3S1358	Chr03		15/16	15/16
TH01	Chr11	Yes	9	9
D21S11	Chr21		29/31	29/31
D18S51	Chr18		13/14	13/14
Penta_E	Chr15		15/20	15/20
D5S818	Chr05	Yes	11/13	11/12
D13S317	Chr13	Yes	9/13	9/13
D7S820	Chr07	Yes	10	10
D16S539	Chr16	Yes	12/13	12/13
CSF1PO	Chr05	Yes	10/11	10/11
Penta_D	Chr21		9/13	9/13
AMEL	X/Y	Yes	X/Y	X/Y
vWA	Chr12	Yes	17	17
D8S1179	Chr08		15/17	15/16
TPOX	Chr2	Yes	8/9	8/9
FGA	Chr04		22/25	22/25

2.2. Electropherogram

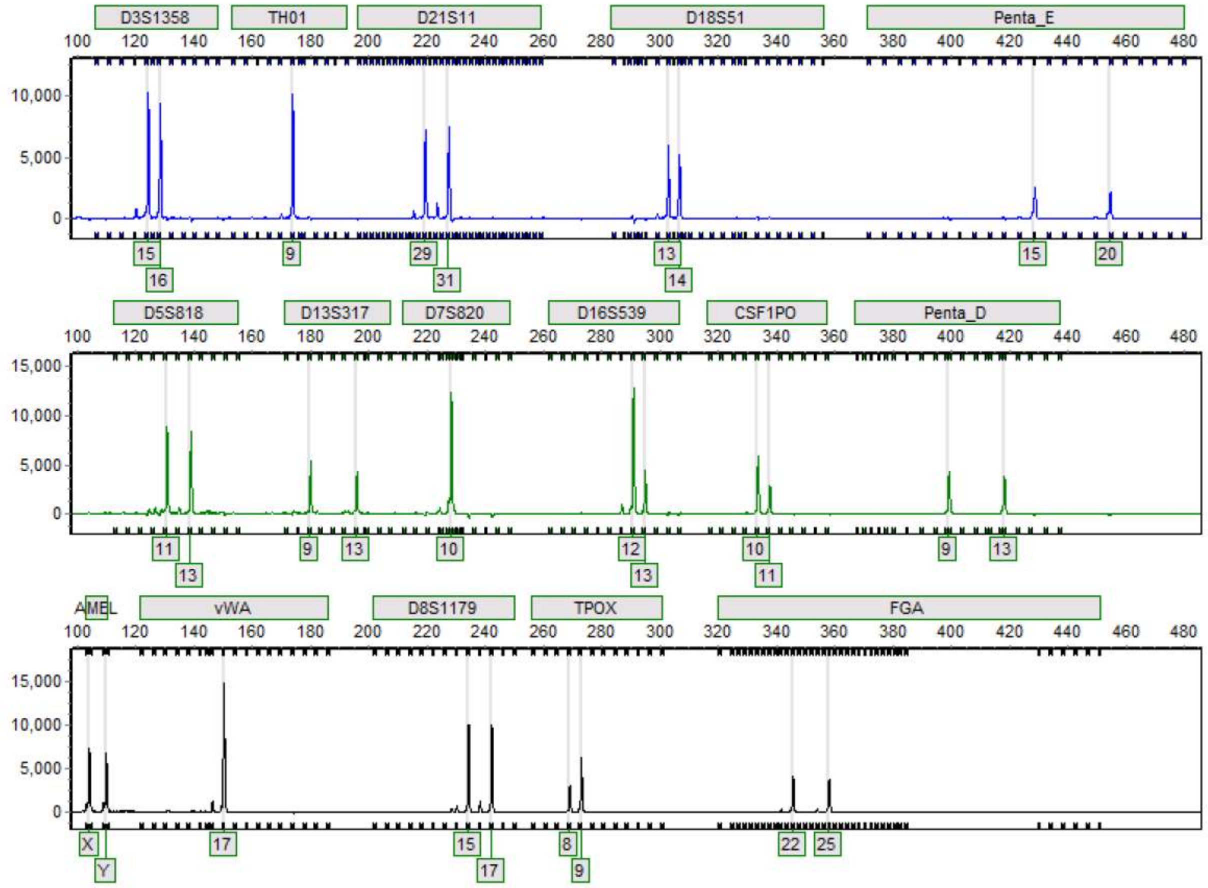

3. Conclusion

According to our analysis of the submitted sample there is no detectable contamination with human origin.

The analyzed data of the submitted sample match 93.3 % to the reference STR profile of the cell line Hep-G2 (Cellosaurus, RRID:CVCL_0027).

4. Glossary

Short Tandem Repeats (STRs)

Short tandem repeats (STRs) consist of a DNA motif of 2-13 bases that are repeated up to several hundred times. The number of repeats in a STR is highly variable among individuals, resulting in fragment length differences if amplified using PCR. These differences in fragment lengths at different loci are used for profiling the cell lines.

Stutter Peaks

Stutter peaks are small peaks which occur immediately before or after the true peak. Stutter peaks are commonly caused by a slippage of the polymerase during the PCR amplification.

Detection of Cell Line Mixtures

Contamination of one cell line by one or several other cell lines can be detected down to a frequency of the contaminating cell line of 10%. Typically, cell line mixtures will result in STR profiles including three or more peaks for single or multiple loci. If CLS notices a possible contamination of a cell line, we will comment the finding in the conclusion part of the analysis.

Peak height ratio

Peak height ratio <25 % (to the highest peak within a STR) is mentioned in the summary table (comments). Peak height ratios <25% need not necessarily influence the behavior or characteristics of the cell line. A small peak height may be due to reduced amplification efficiency, for example resulting from a mutation in the primer site. The reason for the difference in peak heights observed, however, would need some in depth analysis of the test item.

5. General Comment, Confidentiality & Compliance

The results refer only to the portion of the sample CLS has analyzed and might not be assigned unconditionally to the whole sample. This report is the confidential property of the client addressed. All aspects of this study were in accordance with ISO 9001:2015 standards.
